# Capacity of Broadly Neutralizing Antibodies to Inhibit HIV-1 Cell-Cell Transmission Is Strain- and Epitope-Dependent

**DOI:** 10.1371/journal.ppat.1004966

**Published:** 2015-07-09

**Authors:** Lucia Reh, Carsten Magnus, Merle Schanz, Jacqueline Weber, Therese Uhr, Peter Rusert, Alexandra Trkola

**Affiliations:** Institute of Medical Virology, University of Zürich, Zürich, Switzerland; The Scripps Research Institute, UNITED STATES

## Abstract

An increasing number of broadly neutralizing antibodies (bnAbs) are considered leads for HIV-1 vaccine development and novel therapeutics. Here, we systematically explored the capacity of bnAbs to neutralize HIV-1 prior to and post-CD4 engagement and to block HIV-1 cell-cell transmission. Cell-cell spread is known to promote a highly efficient infection with HIV-1 which can inflict dramatic losses in neutralization potency compared to free virus infection. Selection of bnAbs that are capable of suppressing HIV irrespective of the transmission mode therefore needs to be considered to ascertain their *in vivo* activity in therapeutic use and vaccines. Employing assay systems that allow for unambiguous discrimination between free virus and cell-cell transmission to T cells, we probed a panel of 16 bnAbs for their activity against 11 viruses from subtypes A, B and C during both transmission modes. Over a wide range of bnAb-virus combinations tested, inhibitory activity against HIV-1 cell-cell transmission was strongly decreased compared to free virus transmission. Activity loss varied considerably between virus strains and was inversely associated with neutralization of free virus spread for V1V2- and V3-directed bnAbs. In rare bnAb-virus combinations, inhibition for both transmission modes was comparable but no bnAb potently blocked cell-cell transmission across all probed virus strains. Mathematical analysis indicated an increased probability of bnAb resistance mutations to arise in cell-cell rather than free virus spread, further highlighting the need to block this pathway. Importantly, the capacity to efficiently neutralize prior to CD4 engagement correlated with the inhibition efficacy against free virus but not cell-cell transmitted virus. Pre-CD4 attachment activity proved strongest amongst CD4bs bnAbs and varied substantially for V3 and V1V2 loop bnAbs in a strain-dependent manner. In summary, bnAb activity against divergent viruses varied depending on the transmission mode and differed depending on the window of action during the entry process, underscoring that powerful combinations of bnAbs are needed for *in vivo* application.

## Author Summary

When selecting broadly neutralizing antibodies (bnAbs) for clinical application, potency and breadth against free viruses are vital, but additional features may be needed to ensure *in vivo* efficacy. Considering that HIV-1 can utilize free virus and cell-cell transmission to infect, the efficacy of neutralizing antibodies *in vivo* may depend on their ability to block both pathways. While breadth and potency of bnAbs against free viruses have been intensely studied, their precise activity during cell-cell spread remains uncertain. Our analysis of the cell-cell neutralization capacity of a large selection of bnAbs against a spectrum of HIV-1 strains revealed that while bnAbs showed an overall decreased activity during cell-cell transmission, losses varied substantially depending on bnAb and virus strain probed. Although bnAbs occasionally retained activity during cell-cell transmission for individual viruses, this ability was rare and generally not associated with a high potency against free virus spread. Notably, neutralization of free virus but not cell-cell transmission was linked with the activity of bnAbs to inhibit prior to CD4 engagement, highlighting the functional differences of the processes. Since no single bnAb combines the entire range of mechanistic features anticipated to support *in vivo* efficacy, our study adds further evidence that combinations of bnAbs need to be considered for human application.

## Introduction

Recently identified highly potent broadly neutralizing HIV antibodies (bnAbs) are considered as lead components for vaccines and immunotherapeutics (reviewed in [1–5]) and extensive characterization of these bnAbs *in vitro* and *in vivo* is underway to select the most promising candidates [6]. Proof of activity in animal models, the SHIV rhesus macaque infection model or HIV infection of humanized mice, is considered the most conclusive efficacy testing and is required before application in humans can be considered [7–14]. However, investigations in animal models are currently restricted to only few viral strains, limiting the possibility to judge the *in vivo* breadth of the bnAbs tested. Assessment of breadth currently only relies on free virus inhibition *in vitro*, most commonly in the standardized TZM-bl reporter neutralization assay [15,16]. Comparison of bnAb activity *in vitro* and their neutralizing titers *in vivo* has indicated, however, that required doses *in vivo* are >100-fold higher [12,13,17–19] which has been partially attributed to lower tissue concentration of delivered antibodies [11,20], the need to potently elicit antibody-effector functions [21–23] and a reduced activity of antibodies in cell-cell transmission [24–30]. Recent years have broadened our understanding of the HIV infection process and highlighted that the virus has multiple ways of entering and infecting target cells including both, infection by free viruses and direct virus transmission from infected to non-infected cells [31–34]. Cell-cell transmission proved, at least *in vitro*, to be substantially more efficient than free virus spread [35–41] and may hence foster replication of virus strains with low replicative capacity [42]. While the relative impact of free virus and cell-cell transmission *in vivo* remains to be defined, intense research efforts have delineated the molecular processes involved in HIV-1 cell-cell transmission (reviewed in: [43,44]). Aspects of cell-cell transmission have been studied in a range of experimental setups reaching from visualization of individual virological synapses-the contact area that forms between T cells during viral cell-cell transmission- to replication assays in primary cells [25,26,30–33,38,41,45–51]. However, all experimental approaches so far share the difficulty in monitoring true cell-cell transmission events by dissecting these from free virus infection and cell fusion. Assay formats are thus commonly tailored to address individual research questions reducing the possibility for direct comparisons between studies. Cell-cell transmission systems described to date vary in respect to donor and target cells, HIV strains and infection systems (multiple–round and single round infection), virus input and readouts used (reporter assays, direct detection of HIV-1 antigens, single cell and bulk cell analysis) and these differences are thought in part to have led to contradicting observations in respect to neutralizing antibody and ART activity during cell-cell transmission [25–27,46,47,50–54]. Despite these discrepancies, there is agreement that cell-cell transmission, at least *in vitro*, is vastly more efficient than free virus spread and leads to multiple infection of target cells [36–38]. Further, higher rates of transferred viruses seem to reduce the efficacy of reverse transcriptase (RT) inhibitors [46,47,50,51]. While neutralizing antibodies and entry inhibitors with few exceptions displayed reduced efficacy during cell-cell transmission, the magnitude of the reported activity loss varied across different studies [25–27], suggesting that a range of factors contribute to the efficacy of neutralizing antibodies during cell-cell transmission and that not all of these factors are captured equally by the different assay systems. Indeed, functional differences of the entry process [49], the maturation status of the virus during cell-cell transmission [49] and the stage of the entry process blocked by neutralizing antibodies [25,30] have been proposed as factors that influence antibody activity during cell-cell transmission.

To verify bnAb activity during cell-cell transmission, a widely applicable assay such as the TZM-bl neutralization assay used for screening neutralization against free virus infection would be highly desirable. Preferably, such an assay should allow for high throughput inhibitor screening under physiological relevant conditions, most ideally by studying T cell to T cell transmission. Free virus infection (e.g. by using single round replicating viruses) and cell fusion (e.g. by using donor cells that solely express envelope) can be readily assessed *in vitro*. In contrast, monitoring cell-cell transmission while excluding the influence of free virus infection and fusion events remains challenging and is in most assay formats only partially achieved [26,27,46]. In the present study, we adapted the recently established inGluc reporter system [41,55,56] to allow for a side-by-side assessment of bnAb neutralization of free virus infection of T cells and cell-cell transmission to T cells using A3.01-CCR5 T cells as target cells. Neutralization during cell-cell transmission in this assay proved to perform identical to PBMC-PBMC transmission and allowed us to probe 16 bnAbs targeting the CD4bs, the V3 glycan region, the V1V2 loop and the MPER domain in their activity against 11 HIV-1 strains from subtypes A, B and C.

The mechanistic features of bnAb neutralization we explored in the present study may help to explain their *in vivo* function and to select of the most promising candidate bnAbs for therapeutics and vaccine development. Besides probing the capacity of bnAbs to inhibit cell-cell transmission at comparable breadth and potency as free virus spread, we measured the bnAbs’ ability to access the viral envelope (Env) trimer pre- and post-CD4 engagement, as we had previously observed that post-CD4 engagement may be beneficial for maintaining neutralization activity during cell-cell transmission [25].

As we highlight here, bnAbs vary substantially in their capacity to inhibit cell-cell transmission of divergent strains. While we identified bnAbs that retain efficacy to a higher extent, our data reveal that loss of activity during cell-cell transmission cannot generally be predicted by a bnAb’s performance in free virus inhibition. CD4bs- directed bnAbs showed a wide range of activity loss, irrespective of their potency against free virus spread. Unexpectedly, for bnAbs directed to the V3 glycan domain and the V1V2 loop, inhibitory concentrations required for free virus inhibition were inversely correlated with activity loss during cell-cell transmission. This indicates that bnAb features steering high potency to block free virus transmission are not necessarily a driving factor for inhibiting cell-cell transmission. In some cases, V1V2-specific bnAbs displayed even higher activities against cell-cell transmission than free virus infection. Inhibitory activity post-CD4 engagement also differed in a strain- and bnAb-dependent manner and was inversely linked with pre-CD4 attachment activity.

## Results

### Discriminating between free virus infection, cell-cell transmission and cell-cell fusion events

To enable a controlled comparison of bnAb neutralization activity in free virus and cell-cell transmission, we sought to employ an assay format, in which i) both free virus and cell-cell transmission are studied using Env-pseudotyped viruses to limit the infection to a single round, ii) target cells used for free virus and cell-cell transmission are identical and yield results comparable to primary T cell (PBMC) infection, iii) the assays are scalable and can be used to evaluate larger antibody and virus panels.

Considering the potential of the standardized TZM-bl neutralization assay that utilizes 293-T-produced Env pseudoviruses for infection [57], co-culture of TZM-bl with 293-T donor cells expressing Env pseudoviruses would appear as an attractive setup to screen and compare neutralization activity during cell-cell transmission. However, co-cultures of TZM-bl and pseudovirus expressing 293-T cells are highly prone to fusion, saturating the attainable luciferase reporter signal through direct transfer of Tat from donor cells to an extent that *de novo* infection by free virus and cell-cell transmission cannot be reliably quantified simultaneously. While there is certainly interest in exploring cell fusion during the interactions of infected cells in *vivo* [58], we here specifically aimed at comparing the sensitivity of virions during free virus and cell-cell transmission using an assay setup that allows to exclusively monitor these two events. To this end, we employed a recently developed reporter virus system [41,55,56], specifically tailored to record cell-cell transmission using a readout that is not influenced by cell fusion. The system relies on a reporter pseudotyped vector that contains an intron-regulated Gaussia luciferase gene (NLinGluc). Reverse orientation of the Gaussia luciferase gene in the plasmid and the intron prevent luciferase expression from the transfected vector in the producer cells. Gaussia luciferase is only expressed upon successful infection of the target cell by the reporter viruses carrying the intron spliced genomic viral RNA and its reverse transcription. By this, a reporter signal can only result from an infection by free virus or cell-cell transmission but not from cell fusion events or non-productive virus uptake. While the NLinGluc reporter virus has been designed to distinguish cell-cell transmission from fusion, it needs to be combined with measures that restrict free virus transmission during co-culture if genuine cell-cell transmission needs to be assessed. As recently described [25], we made use of the fact that for many HIV strains efficient infection of transformed cell lines by free viruses but not cell-cell transmission requires the addition of polycations such as DEAE [16,25,59–62]. Polycation dependency is most prominent for R5 viruses with a low positive V3 net charge. These viruses have a decreased capacity to overcome the charge repulsion of negatively charged membrane proteins of the viral and cellular membrane which appears to be infection limiting in transformed cell lines [60–63]. Importantly, as previously shown, neutralization efficacy is not affected by the presence or absence of polycations [60–63].

**Fig 1:**
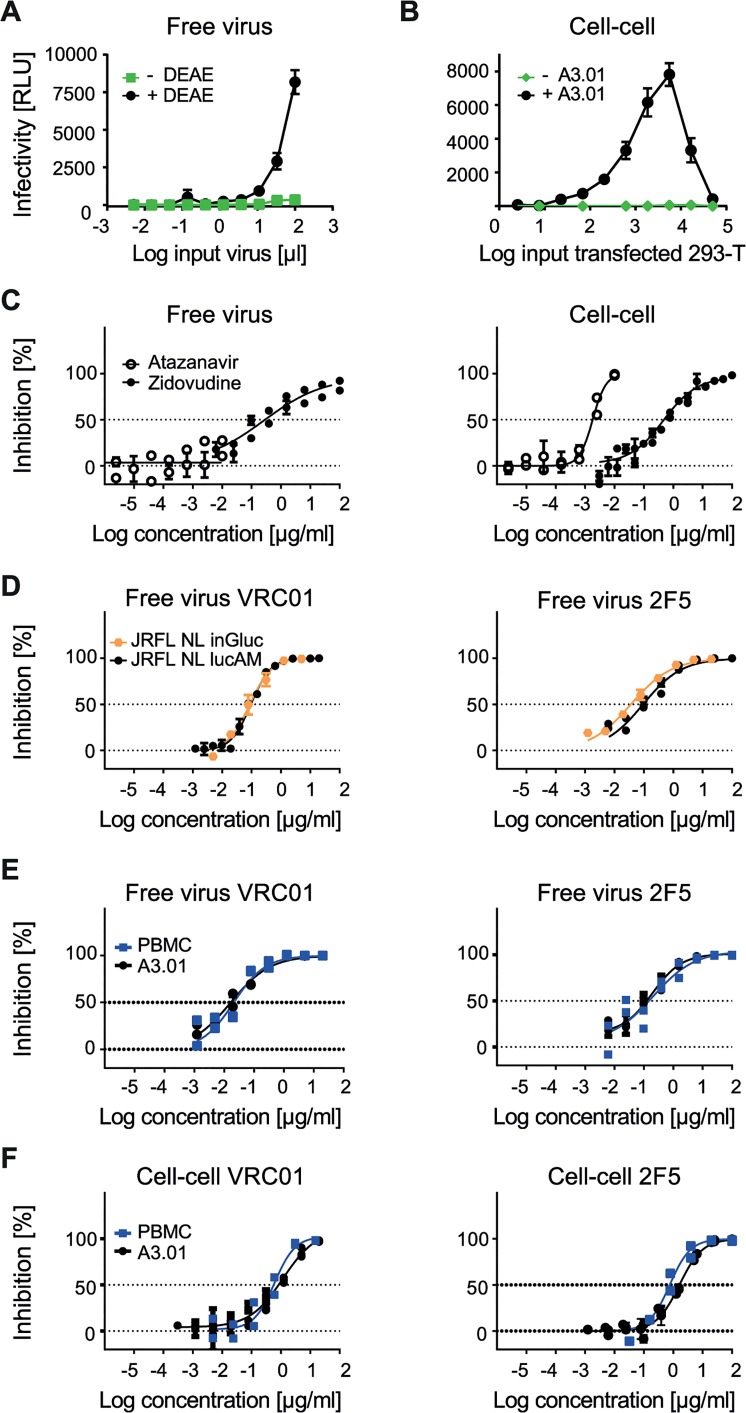
Evaluating A3.01-CCR5 T cell-based free virus and cell-cell transmission. **A to F:** As indicated, the Gaussia Luciferase reporter virus NLinGluc and the firefly luciferase reporter virus system NLlucAM were used to study free virus and cell-cell transmission. Infectivity was measured by assessing Gaussia and firefly luciferase reporter activity in the supernatant and from the lysed cells, respectively. The graphs show means and standard error of means (SEM, error bars) of two to three independent experiments performed in duplicates. Panels C-F depict curve fits to sigmoid dose response curves (variable slope). **A: DEAE dependency of the NLinGluc reporter virus.** JR-FL NLinGluc free virus was titrated on TZM-bl cells in 96-well plates in presence (black circles) or absence (green squares) of 10 µg/ml diethylaminoethyl (DEAE). **B: Absence of Gaussia luciferase activity in 293-T donor cells but high signal in 293-T-A3.01-CCR5 co-cultures allows for specific detection of cell-cell transmission.** 293-T cells transfected with JR-FL *env* and NLinGluc reporter virus were titrated and incubated in presence of 1.5*10^4^ A3.01-CCR5 target cells per 96 plate culture well (+A3.01, black circles) or in medium alone (-A3.01, green diamonds) in the absence of DEAE. **C: Assessing the sensitivity to antiretrovirals to validate free virus and cell-cell assays.** Testing the sensitivity of A3.01-CCR5 infection by free virus (JR-FL NLlucAM firefly luciferase reporter virus; left) and cell-cell (JR-FL NLinGluc transfected 293-T; right) transmission to the RT inhibitor Zidovudine (black circles) or the protease inhibitor Atazanavir (open circles) to verify that both pathways are sensitive to RT inhibition but only cell-cell transmission is affected by protease inhibition. **D: NLinGluc (Gaussia) and NLlucAM (firefly) luciferase reporter viruses yield comparable results in free virus inhibition assays.** Free virus inhibition of JR-FL NLinGluc (yellow circles) and JR-FL NLlucAM (black circles) reporter viruses by bnAbs VRC01 and 2F5 was compared. For both viruses, 100% infectivity was determined in cultures without inhibitor. **E: Equal neutralization sensitivity of free virus infection on A3.01-CCR5 and PBMC target cells.** Inhibition of free virus infection of PBMC (blue squares) or A3.01-CCR5 (black circles) by bnAbs VRC01 and 2F5 was studied using the firefly reporter virus JR-FL NLlucAM. For both cell types 100% infectivity was determined in cultures without inhibitor. **F: Equal sensitivity to neutralization in 293-T-A3.01-CCR5 and PBMC-PBMC cell-cell transmission.** The capacity of bnAbs VRC01 and 2F5 to block cell-cell transmission was assessed in co-cultures of JR-FL NLinGluc-transfected 293-T with A3.01-CCR5 (black circles) and JR-FL infected PBMC with rhTRIM5α-transduced PBMC (blue squares). Infectivity was assessed via determination of Gaussia luciferase activity in the 293-T-A3.01-CCR5 co-cultures or via intracellular p24 staining and flow cytometry analysis for the PBMC co-cultures. For both co-cultures, 100% infectivity was determined in cultures without inhibitor.

Since T cell to T cell transfer is considered of high relevance for HIV-1 spread *in vivo* [64], we chose the T cell line, A3.01-CCR5, as target cells for free virus and cell-cell transmission. In a first step, we verified that free virus infection with Env-pseudotyped NLinGluc reporter viruses, like the commonly used reporter virus NLlucAM [65] which encodes for firefly luciferase, requires DEAE for efficient infection of A3.01-CCR5 T cells (Fig 1A, S1 Fig). This ensures that by omission of DEAE during co-culture, we only allow for HIV cell-cell transmission but not free virus infection to occur [25]. While 293-T donor cells transfected with Env-pseudotyped NLinGluc reporter viruses generated no Gaussia luciferase signal (Fig 1B), co-culture of these donor cells with A3.01-CCR5 target cells in absence of DEAE yielded Gaussia luciferase activity, indicating cell-cell transmission (Fig 1B). To further verify that the measured reporter activity was indeed due to genuine cell-cell transmission and not influenced by free virus infection or cell fusion, we studied the effect of reverse transcriptase (RT) and protease inhibition on cell-cell and free virus transmission. Free virus spread was vulnerable to RT inhibition but not protease inhibition, as virions in the harvested virus stocks are fully matured. In contrast, cell-cell transmission proved fully sensitive to both RT and protease inhibitors as described, excluding a reporter gene transfer by fusion [47,51] (Fig 1C).

### Identical neutralization patterns during PBMC to PBMC transmission and 293-T to A3.01-CCR5 transmission

The choice of donor and target cells for cell-cell transmission assays suitable for neutralization screening is not trivial as the selected system should provide the most physiologically relevant information, most ideally by using primary T cells, but at the same time guarantee high intra- and inter-assay reproducibility, high throughput capacity and cost effectiveness. To verify our choice of A3.01-CCR5 cells as target cells, we first compared neutralization of free virus infection of A3.01-CCR5 cells using the JR-FL-pseudotyped NLinGluc (Gaussia) and NLlucAM (firefly) reporter viruses. Infection with both virus constructs yielded identical neutralization profiles for all bnAbs tested (Fig 1D, S2 Fig) and thus allowed us to use the less expensive and more stable luminescence signal emitting firefly reporter readout for the assessment of free virus neutralization on A3.01-CCR5.

The cell-cell transmission system we employed here, utilizes 293-T cells as donor cells as these cells, unlike T cells, can be transfected with high efficiency and are thus commonly used to produce HIV-1 pseudoviruses. As therefore in our cell-cell transmission setup only one of the partners, the A3.01-CCR5 target cells, are T cells, we sought to verify if the transmission from 293-T to A3.01-CCR5 is comparable to genuine T cell to T cell transmission and yields similar neutralization patterns. To this end, we studied HIV-1 inhibition by a selection of bnAbs during free virus infection of PBMC and cell-cell transmission of PBMC to PBMC, as PBMC are considered the most physiological relevant *in vitro* cell system available. Of note, assessment of free virus inhibition using a range of Env-pseudotyped NLlucAM (firefly) reporter viruses on PBMC and A3.01-CCR5 yielded almost identical inhibitory patterns, indicating that A3.01-CCR5 cells are indeed a valid substitute for PBMC (Fig 1E, S3 Fig). Inhibition of PBMC to PBMC transmission was studied using JR-FL and JR-CSF infected PBMC as donor cells and rhTRIM5α overexpressing PBMC as target cells in which free virus infectivity is restricted [25]. Infection in the PBMC co-cultures was assessed by monitoring Gag protein transfer from infected PBMC to rhTRIM5α expressing PBMC target cells by flow cytometry as previously described [25]. Most notably, the PBMC-PBMC and the 293-T-A3.01-CCR5 neutralization assays yielded identical inhibitory profiles (Fig 1F, S4 Fig), confirming that the 293-T to A3.01-CCR5 transmission assay has the capacity to capture the essential components of PBMC-PBMC transmission in respect to antibody accessibility and activity during cell-cell transmission. Based on this, we concluded that A3.01-CCR5 T cells, albeit no primary T cells, are valid target cells for the cell-cell transmission studies.

### Determining the breadth and potency of bnAbs in inhibiting cell-cell transmission

A main goal of our study was to assess the efficacy of bnAbs during cell-cell compared to free virus transmission to derive insights into the magnitude of the neutralization activity losses during cell-cell transmission, as this might be important information when using bnAbs as therapeutics or components of vaccine induced immunity.

To assess the breadth of bnAb activity during cell-cell transmission, we sought to test a range of genetically different virus strains as so far most cell-cell neutralization studies have included only a comparatively small number of viruses, mostly from subtype B [25–27]. Our virus panel consisted of 11 viruses from subtype A, B, and C and from different disease stages (transmitted founder (T/F) viruses, acute and chronic infection; S1 Table). BnAbs that were probed for activity in free virus and cell-cell transmission included bnAbs directed to the CD4bs (b12, VRC01, NIH45-46, PGV04, 3BNC117), the V3-glycan region (PGTs 121, 125, 128, 135), V1V2-glycan dependent PGT145, PG9, PG16) and the MPER domain (2F5, 10E8, 4E10) (S2 Table).

**Fig 2:**
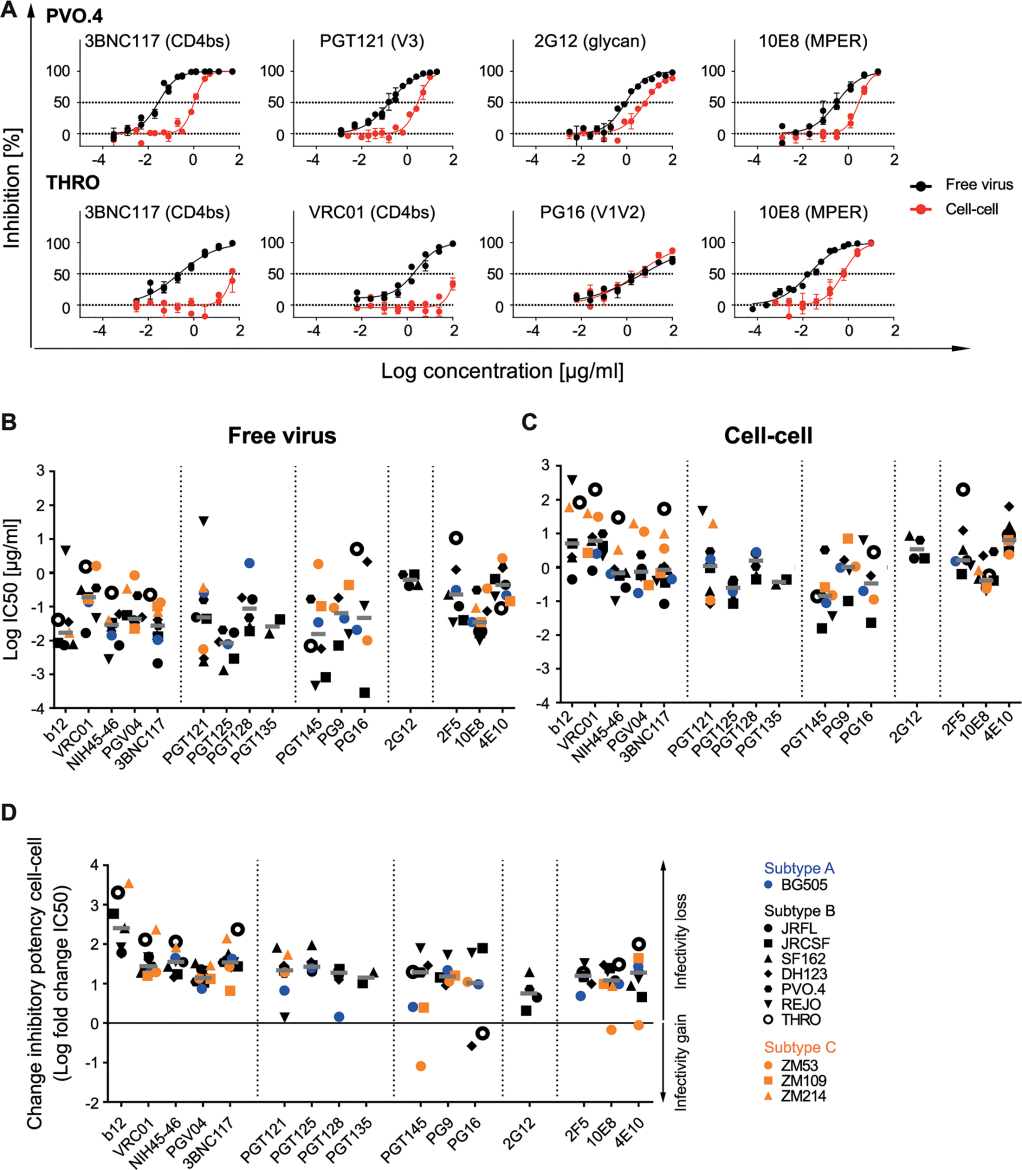
Decrease of bnAb activity during cell-cell transmission is variable and strain-dependent. **A: Diverse pattern of bnAb activity loss within and across viral strains.** Inhibition of free virus (black circles) and cell-cell (red circles) transmission of subtype B virus strains PVO.4 and THRO by the indicated bnAbs was studied. The graphs show means and standard error of means (SEM, error bars) of two to three independent experiments performed in duplicates and curve fits to sigmoid dose response curves (variable slope). **B, C: Inhibitory activity of bnAbs in free virus and cell-cell transmission of divergent virus subtypes.** 50% inhibitory concentrations (IC_50_ in µg/ml) of bnAbs against a panel of subtype A (blue), B (black) and C (orange) virus strains in free virus **(B)** and cell-cell **(C)** transmission. Graphs depict IC_50_ values of all bnAb-virus pairs for which the virus proved sensitive. Non-sensitive combinations are recorded in S3 Table. Grey bars depict median IC_50_ values for all virus strains sensitive to a given bnAb. **D: bnAbs predominantly lose activity in cell-cell transmission.** The change in inhibitory activity in cell-cell transmission compared to free virus inhibition is expressed as the ratio of IC_50_ cell-cell/IC_50_ free virus (fold change IC_50_). Subtype A, B and C viruses are denoted in blue, black and orange, respectively. A fold change IC_50_ of 0 indicates an equal activity during free virus and cell-cell transmission (black line). Median fold change IC_50_ (grey bars) for all virus strains sensitive to a given bnAb are shown.

When we assessed the activity loss during cell-cell transmission for the seven subtype B viruses included in our panel, we observed a strong decrease in neutralization capacity during cell-cell transmission for the majority of bnAbs (Fig 2A, S5A–S5P Fig and S6A Fig) confirming earlier findings [25–27]. The loss in bnAb activity occurred independent of the epitope region targeted or the specific bnAb analyzed and its extent varied substantially for individual bnAbs against the divergent viruses. Further, the decrease in neutralization activity was not specific to the virus strains tested. As exemplified for strain PVO.4, the highest loss in activity was seen for bnAb 3BNC117 with a 34-fold increased 50% inhibitory concentration (IC_50_) during cell-cell transmission whereas bnAb 2G12 retained its activity at higher levels with only a 7.2-fold loss in activity (Fig 2A, S3 Table). Across viruses and bnAbs tested, we observed an overall diverse pattern. Intriguingly, some rare bnAb-virus pairings showed no reduction in activity during cell-cell transmission as exemplified by PG16 neutralization of strain THRO (Fig 2A). This is particular notable as other bnAbs, the CD4bs (b12, VRC01, NIH45-46 and 3BNC117) probed in parallel against the same isolate, showed a pronounced decrease in activity during cell-cell transmission (100–2000-fold increased IC_50_; Fig 2A, S3 Table).

Overall, the individual potencies of the bnAbs against the probed subtype B viruses varied substantially in both, the free virus and the cell-cell transmission setting. To probe if the observed decrease in neutralization activity is subtype-specific or a general feature of HIV-1 cell-cell transmission, we expanded our analysis to investigate viruses from subtype A (BG505) and Subtype C (ZM53, ZM109, ZM214) (Fig 2B, 2C and S6A Fig). Free virus and cell-cell transmission of these viruses proved to follow the same pattern as we had observed for subtype B viruses with bnAb potency against individual viruses varying in a wide range for both transmission routes (Fig 2B and 2C and S6A Fig).

To compare the extent of change in inhibitory activity during cell-cell transmission, we determined the fold changes in IC_50_ (Fig 2D, S6B and S6C Fig). Across bnAbs, the median IC_50_ against all strains was elevated for cell-cell compared to free virus transmission (Fig 2B and 2C, S3 Table), resulting in a 4.5-fold loss in activity for bnAb 2G12 to 256-fold loss for bnAb b12 with an overall median fold reduction of 22. Although few in numbers, some bnAb-virus combinations yielded activity in cell-cell transmission inhibition that closely matched their potency against free virus (less than 5-fold reduced; Fig 2D). These were 2G12 (with strains JR-FL and JR-CSF), PGT121 (with strains REJO and BG505), PGT128 (with strain BG505), PGT145 (with strains BG505 and ZM109), 10E8 (with strain ZM53) and 4E10 (with strains JR-CSF and ZM53). Most intriguingly, PGT145 neutralized ZM53 better during cell-cell transmission (12-fold lower IC_50_). A similar trend was seen for PG16 against strains DH123 and THRO although there the increases in activity during cell-cell transmission were smaller. Apart from the two MPER bnAbs, all other bnAbs which occasionally yielded identical activity in free virus and cell-cell neutralization had glycan-dependent epitopes centering in or around the V3 loop (PGT121, PG128, 2G12) or V1V2 loop (PGT145, PG16). In contrast, among CD4bs-directed bnAbs for none of the probed virus strains identical activity during cell-cell transmission was obtained (S5A–S5E Fig). BnAb PGV04 retained neutralization activity during cell-cell transmission to the highest extent, while b12, the weakest and least broad of the probed CD4bs bnAbs, completely lost activity against several viruses (S5A Fig).

Of note, for all epitope classes, the overall median activity loss of the probed bnAbs during cell-cell transmission was in a similar range, irrespective of the virus subtype (S6B Fig). Individual virus strains varied, however, substantially to the extent by which cell-cell transmission affected the inhibitory potentials of bnAbs (S6C Fig).

**Fig 3:**
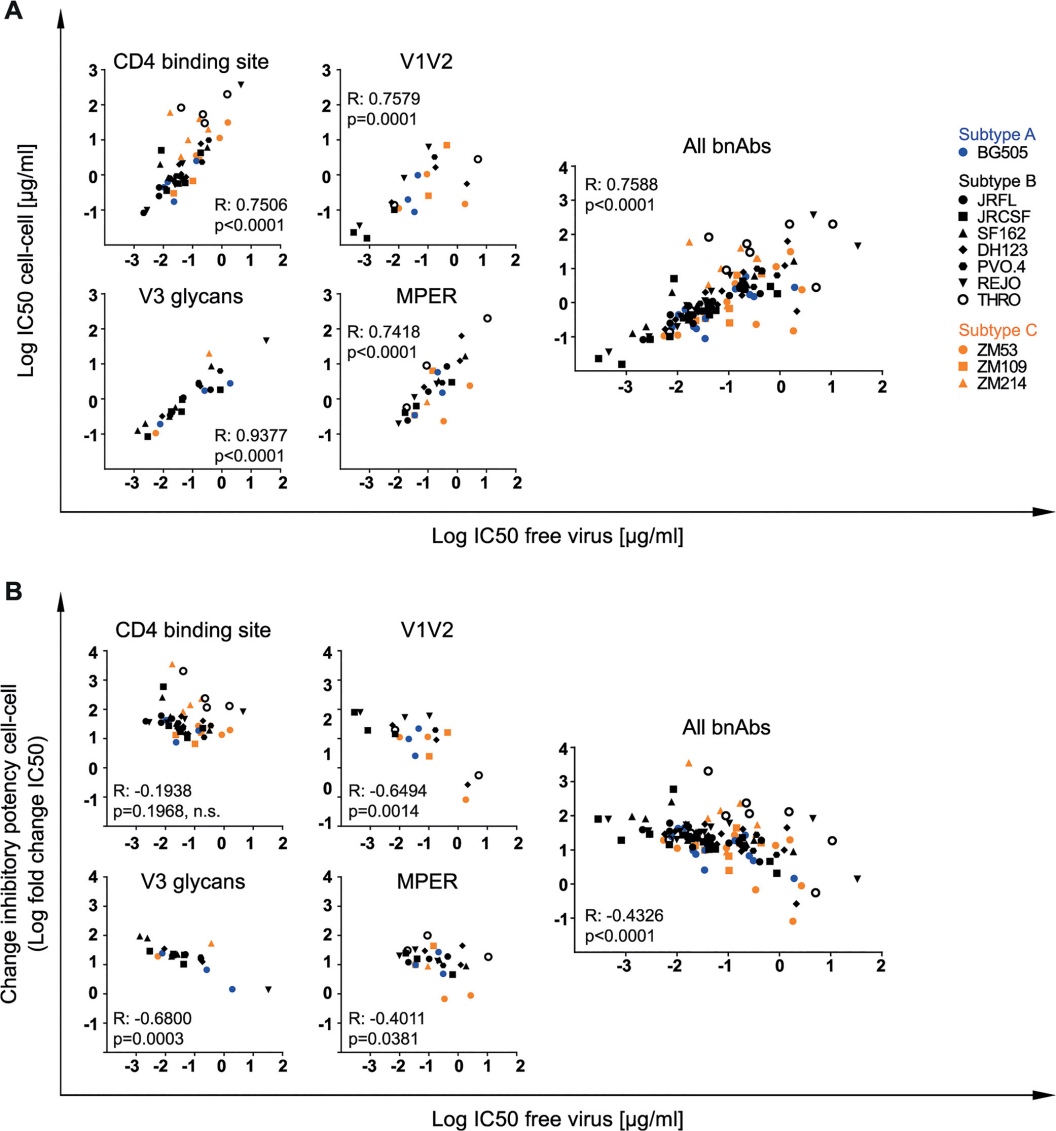
Differential loss in cell-cell neutralization across bnAb classes. Interdependencies of inhibitory activity (IC_50_) against free and cell-cell transmitted virus **(A)** and the fold change in IC_50_ between free and cell-cell transmission **(B)** was determined for individual bnAb classes and all classes combined by Spearman correlation based on the untransformed data sets. R and p values are depicted. n.s. indicates a non-significant correlation. Data are derived from Fig 2, S5A–S5P Fig and S3 Table. Subtype A, B and C viruses are denoted in blue, black and orange, respectively.

To determine if the reported losses in neutralization activity of cell-cell spread can be predicted by the bnAbs’ performance during free virus inhibition or if they need to be determined individually, we assessed the interdependence of potency in free virus and cell-cell neutralization and the extent of the activity loss inflicted by cell-cell transmission (Fig 3A and 3B). Inhibitory concentrations obtained for free virus and cell-cell neutralization proved to be linked (Fig 3A). Unexpectedly, efficacy in free virus inhibition and the extent of activity loss during cell-cell transmission showed an inverse correlation for V1V2- and V3-directed and to a lesser extent also for MPER-directed bnAbs, but not for bnAbs targeting the CD4bs (Fig 3B). Thus, with the exception of CD4bs-directed bnAbs, a higher loss in neutralization activity during cell-cell transmission was more frequently observed when bnAb activity against free virus transmission was very high, suggesting that features which steer the bnAbs’ ability to potently inhibit free virus spread are not equally decisive in cell-cell transmission. In fact, the opposite appeared to be true for some bnAb-virus combinations: The bnAbs that comparably neutralized a virus strain during both transmission modes were in most cases relatively weak inhibitors of these strains (2G12 for JR-FL and JR-CSF, PGT121 for REJO and BG505, PGT128 for BG505, PGT145 for ZM53, PG16 for DH123 and THRO, 10E8 for ZM53, 4E10 for JR-CSF and ZM53). High potency against free virus activity was only in two cases (PGT145 for BG505 and ZM109) paired with low loss of activity during cell-cell transmission (Fig 2B and 2D, S3 Table, S5J Fig). Collectively, this suggests that antibody features that steer high potency against free virus spread are not the driving factors determining the activity during cell-cell transmission.

In summary, the activities of bnAbs during inhibition of free virus and cell-cell transmission proved highly diverse and varied in a strain- and antibody-dependent manner (Fig 2A–2D, S3 Table, S5A–S5P Fig and S6 Fig). None of the 16 probed bnAbs retained the free virus inhibitory activity during cell-cell transmission across all viruses tested, highlighting that the activity loss during cell-cell transmission needs to be considered when determining bnAb breadth and exploring *in vivo* infective doses for bnAb use in therapy and prevention. Amongst the panel of bnAbs probed against the 11 viruses, the CD4bs bnAb PGV04 proved the most consistent and combined high breadth (10 of 11 virus strains inhibited in both transmission modes), potency (median IC_50_ of 0.039 and 0.645 µg/ml for free and cell-cell transmission respectively) and a relatively low loss in activity during cell-cell transmission (median 13-fold reduction, IC_50_ values ranging from 7.5 µg/ml for BG505 to 33 µg/ml for SF162) (Fig 2B–2D, S3 Table, S5C Fig). However, several bnAbs performed in a comparable range: 3BNC117 was closest to PGV04 in performance but displayed a somewhat wider range in activity loss during cell-cell transmission (median 34-fold IC_50_ reduction, range 6.6 for ZM109 to 237 for THRO) (Fig 2B–2D, S3 Table, S5E Fig). The loop and glycan specific, gp120-directed bnAbs probed had a generally lower breadth and hence could not be assessed for the entire virus panel. Amongst these, PGT121 combined best breadth, potency and a low loss during cell-cell transmission. This was also true for the MPER-specific bnAb 10E8 which was very consistent in its activity during both transmission modes, neutralizing all 11 probed virus isolates.

### The fusion inhibitor T-20 outperforms bnAbs in potency and retaining activity during cell-cell transmission

**Fig 4:**
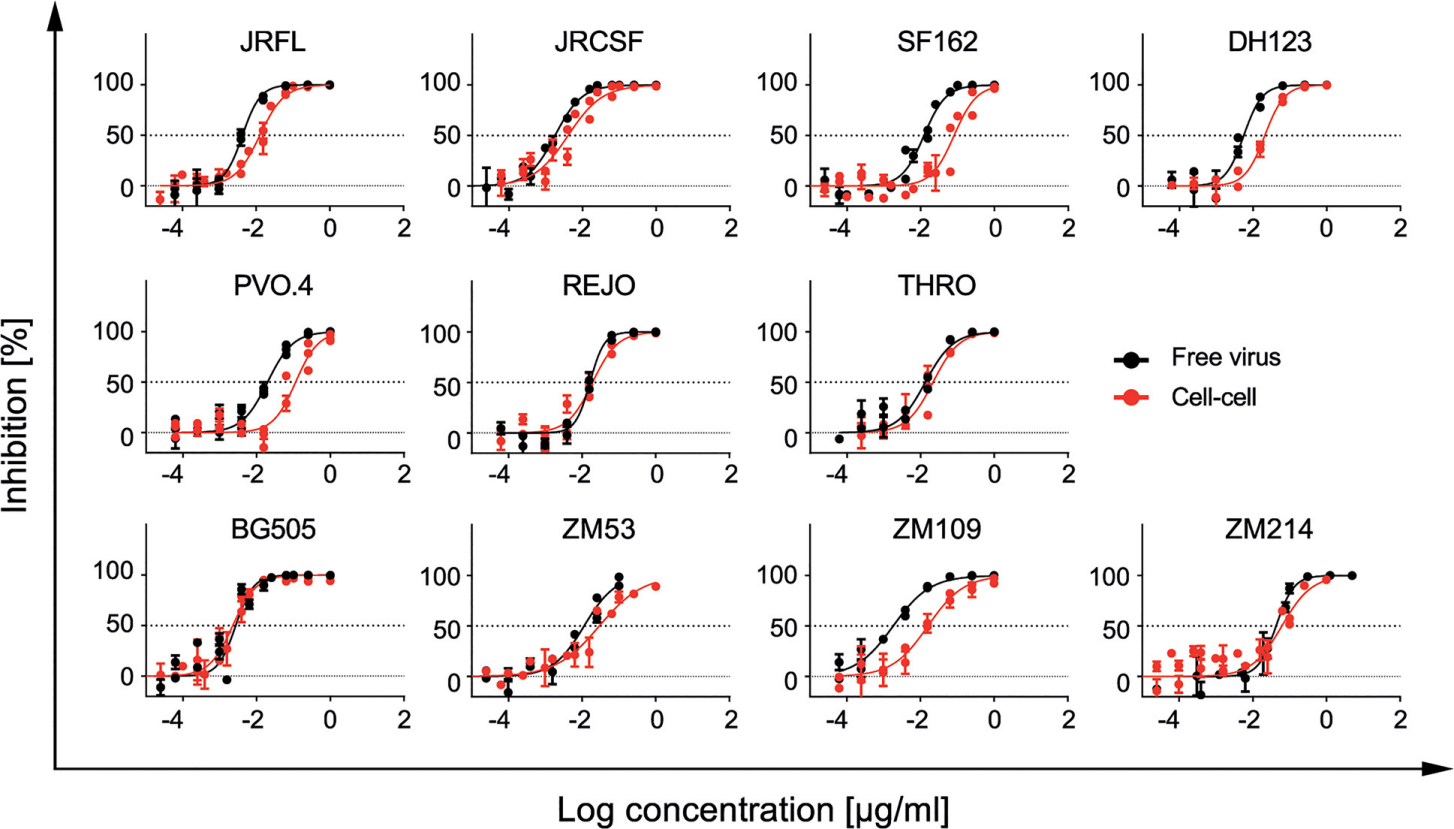
T-20 potently inhibits free virus and cell-cell transmission across diverse virus strains. Inhibition of free virus (black circles) and cell-cell (red circles) transmission of subtype A, B and C viruses by T-20 is shown. The graphs show means and standard error of means (SEM, error bars) of two to three independent experiments performed in duplicates and curve fits to sigmoid dose response curves (variable slope).

To validate the results obtained in the cell-cell transmission studies, we included the gp41-targeting fusion inhibitor T-20 (S2 Table) as a control in all experiments as it has high activity across different genetic subtypes of HIV-1 [66] and our previous work [25] indicated that T-20 has a high capacity to preserve its activity during free virus and cell-cell neutralization. Since T-20, like neutralizing antibodies, targets the virus envelope, we reasoned that T-20 may provide a valuable reference for neutralizing activity in free virus and cell-cell transmission. T-20 proved to be the most successful agent in preserving activity during cell-cell transmission across all virus strains tested (Fig 4, S3 Table). Reduction in activity during cell-cell transmission was less than 10-fold for all 11 probed viruses including strains THRO and ZM214 (median fold reduction for T-20 in cell-cell transmission of 1.6 and 5.3 respectively) which were the most resistant viruses to bnAb neutralization during cell-cell transmission (S3 Table). Thus far, a similarly retained potency during cell-cell transmission has only been reported for cell-directed inhibitors targeting CD4 and co-receptors but not for Env-specific agents [25,27]. Considering that T-20 has been in clinical use [67] and extensive *in vivo* efficacy data are available, our observations highlight that T-20 could be considered as valuable control in future experiments which aim to dissect if a preserved cell-cell transmission activity is required for the *in vivo* efficacy of neutralizing antibodies and entry inhibitors.

### Exploring the influence of efficacy loss during cell-cell transmission on neutralization escape

**Fig 5:**
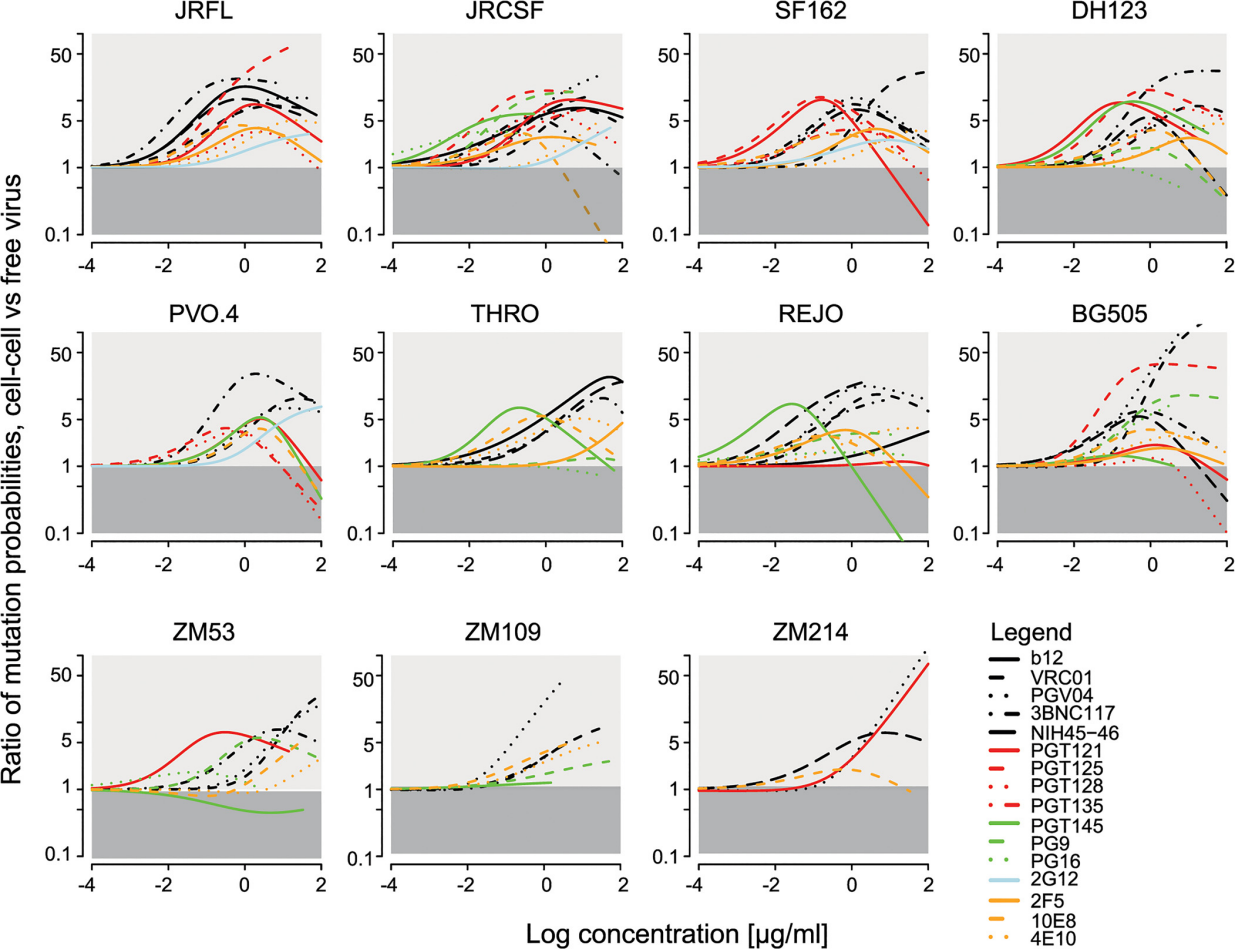
Comparison of bnAb escape mutant occurrence probabilities via cell-cell versus free virus transmission. Graphs display how much more likely it is that an escape variant arose via the cell-cell pathway in comparison to free virus spread. The analysis was performed for all tested bnAb-virus pairs. Light grey areas indicate the concentrations for which an escape variant is more likely to be formed during cell-cell spread. The dark grey areas correspond to the concentration range that favours formation during free virus spread.

A consequence of the activity loss of neutralizing antibodies during cell-cell transmission that needs to be considered is that escape to neutralization may occur more rapidly than via free virus transmission as postulated for reverse transcriptase inhibitors [46]. To determine how much more likely it is that an antibody neutralization resistant variant arose during infection via cell-cell transmission than via free virus spread, we translated this question into a mathematical model (Materials and Methods). To this end, we first derived the mutant occurrence probability for the two pathways, i.e. the probability that an antibody resistant mutant arises depending on the infection pathway. This expression incorporates the measured IC_50_ and slope values (S3 Table and S4 Table) of the bnAb-virus combinations tested in free virus and cell-cell neutralization (S5A–S5P Fig). In a second step, we divided the mutant occurrence probability for cell-cell transmission by the one for free virus transmission. By doing so, the probability that a point mutation arises during reverse transcription, which cannot be experimentally determined, cancels out. This allowed us to determine how much more likely it is that a given mutant arose during cell-cell compared to free virus transmission. Fig 5 depicts the ratios of the mutant occurrence probabilities in dependence of the bnAb concentration. We found that irrespective of the bnAbs’ individual neutralization capacities across a wide range of bnAb concentrations, it is more likely that a neutralization resistance conferring mutation evolves via the cell-cell transmission route (indicated by the light grey shaded area in Fig 5) than via the free virus route (dark grey shaded area). In certain cases (e.g. for bnAb VRC01 and BG505, PGV04 and BG505 and ZM214, PGT121 and ZM214 and PGT125 and JR-FL) probabilities for mutants to occur via cell-cell transmission were up to 50 times higher.

For the analysis, we assumed that the per site mutation probability is the same for cells infected during the cell-cell and free virus pathway. However, as cells infected via cell-cell transmission appear to be more frequently infected with more than one virus [37,38,46], the total number of possible mutations that can occur is higher and recombination of viral RNA from different strains is more likely, which would result in higher diversity. In our experimental setup, this should not be a driving factor as we utilized Env-pseudotyped molecular clones. This may, however, play a role *in vivo* when genetically diverse virus populations are present. We thus tested which influence higher mutation rates in cells infected via cell-cell spread could have on mutant generation. We found that higher mutation rates and diversity in cell-cell transmission would further increase the probability that a mutation arose via cell-cell in comparison to free virus transmission (S7 Fig). Together, these findings support the hypothesis that cell-cell transmission of virus particles can serve as a rescue route from neutralizing antibodies due to the reduced sensitivity of this pathway to neutralization and additionally by fostering the evolution of resistant strains.

### Exploring functional characteristics of bnAbs

**Fig 6:**
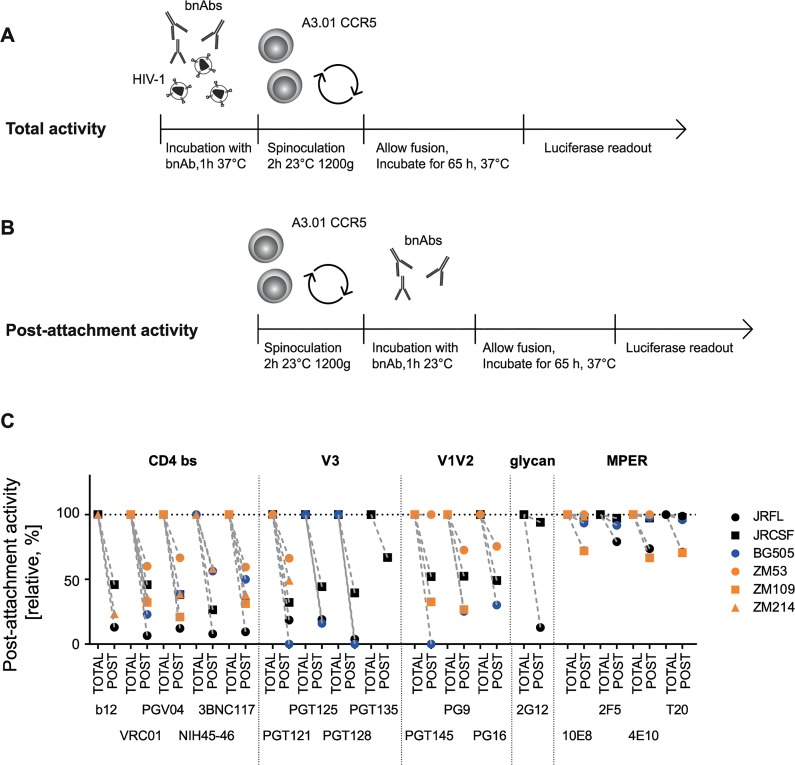
The capacity to neutralize post-CD4 receptor engagement differs between bnAbs and virus strains. **A, B:** Schematic of free virus infection of A3.01-CCR5 target cells to measure total and post-attachment activity of bnAbs. **C:** Total activity of each bnAb-virus combination (where bnAbs were present throughout the infection process) was set to 100% and post-attachment inhibition displayed relative to it. Subtype A, B and C viruses are denoted in blue, black and orange, respectively. Mean values of two to three independent experiments are shown.

Depending on the accessibility of their epitopes during entry, bnAbs differ in their ability to neutralize HIV prior to or post-CD4 engagement [25,68,69]. As discussed previously, the capacity to access the virus post-CD4 interaction may be advantageous for antibodies, particularly in the setting of cell-cell transmission as this may elongate the bnAbs’ window of action [25]. To probe the capacity of bnAbs to interfere with HIV entry pre- and post-CD4 attachment, we first assessed the activity of all 16 bnAbs and T-20 during free virus neutralization of six virus strains from subtypes A, B and C when present during the entire infection period (Fig 6A) or solely added post-CD4 attachment (Fig 6B). To measure total neutralization activity (cumulative bnAb action before and after CD4 attachment; Fig 6A), virus and bnAbs were preincubated before the inocula were spinoculated onto A3.01-CCR5 target cells to synchronize infection. BnAbs were thus present during the entire infection process and could act both, before and after CD4 attachment. Concentrations of bnAbs were chosen to yield maximal inhibition for all viruses in these experiments (S5 Table). As we previously showed, attachment to cells during spinoculation is highly CD4-driven [25], hence allowing a synchronized initiation of infection in our experimental design, starting from CD4-bound virions. To assess their post-attachment inhibition potential, bnAbs were added after spinoculation of viruses onto target cells in identical concentrations (Fig 6B, S5 Table). The inhibitory activity measured post-attachment was expressed relative to the total activity of the bnAb over the entire infection period (Fig 6A) which was set to 100% (Fig 6C). In line with the enhanced accessibility of the MPER domain following receptor binding and rearrangements [70,71], the MPER-directed bnAbs showed high activities when added post-attachment, yielding 100% inhibition in the majority of cases. The lowest post-attachment activities observed for MPER bnAbs were still comparatively high with levels above 67% observed against JR-FL (2F5 79% and 4E10 74%) and ZM109 (10E8 72% and 4E10 67%). Consistent with their epitope specificity, CD4bs-directed bnAbs, on the other hand, only reached post-attachment inhibition capacities which were below 50% for most virus strains. Strain ZM53 was the only exception, as most CD4bs-directed bnAbs retained around 60% of their activity. Post-attachment activity of V3 loop-directed bnAbs was comparable to CD4bs-directed bnAbs and for most bnAb-virus combinations below 50%. The V1V2-directed bnAbs displayed a higher strain-dependency: ZM53, which retained high sensitivity to CD4bs-directed bnAbs at the post-attachment stage, remained fully sensitive to PGT145 and also highly sensitive to PG9 (73%) and PG16 (76%) (Fig 6C). Overall, the variability in post-attachment activity was higher than we previously anticipated, judging from the analysis of smaller antibody and virus panels [25].

**Fig 7:**
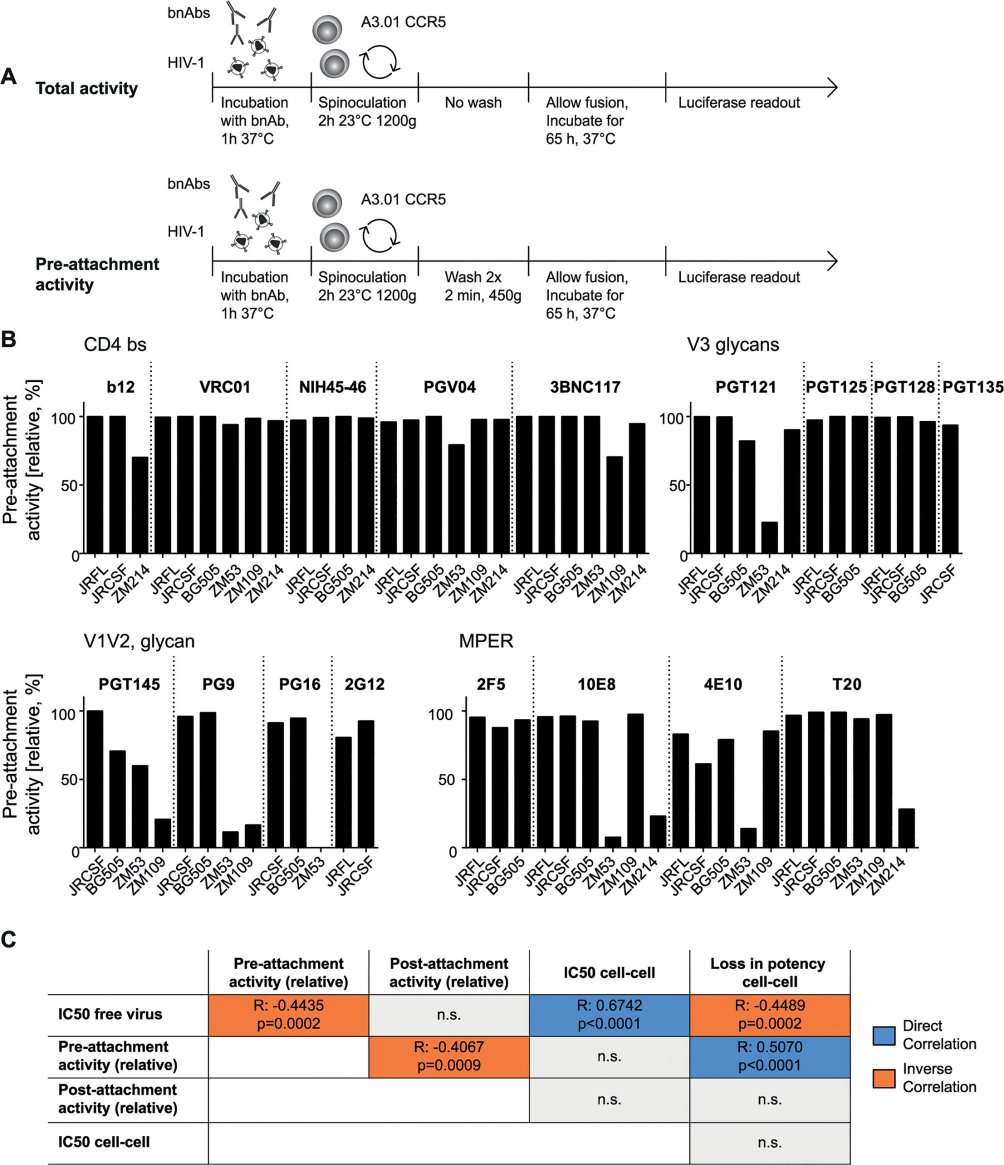
The capacity to neutralize free viruses prior to completion of CD4 attachment varies across bnAb classes. **A:** Schematic of free virus infection of A3.01-CCR5 target cells to measure total and pre-attachment activity of bnAbs. Total activity of each bnAb-virus combination (where bnAbs were present throughout the infection process) was set to 100% and pre-attachment inhibition displayed relative to it. To assess pre-attachment activity, bnAbs were removed following attachment by spinoculation. **B: Pre-attachment neutralization activity is strain- and epitope-dependent.** Pre-attachment activity (relative, %) of the bnAb classes CD4bs, V3 glycans, V1V2 loop, MPER was assessed against the indicated virus strains. Data are means of two to three independent experiments. **C: Pre-attachment activity correlates with high potency against free virus, decreased post- attachment inhibition capacity and loss in activity during cell-cell transmission.** Summary of the interdependencies of pre-attachment, post-attachment inhibition, IC_50_ for free virus inhibition, IC_50_ for cell-cell inhibition and loss in activity during cell-cell inhibition. Correlations were determined by Spearman correlation based on the untransformed data sets and are shown in S8 Fig. R and p values are depicted. n.s. indicates a non-significant correlation. Direct correlation is denoted in blue, inverse correlation in orange.

Comparing total and post-attachment activity additionally provided insight into the pre-attachment activity of the bnAbs, which was measured as the activity against the unbound virus (during pre-incubation) and the initial steps of CD4 engagement (during spinoculation) in our experimental setup (Fig 7A). While a low post-attachment activity implies that a bnAb preferentially acts prior to CD4 engagement, the reverse is not necessarily the case as action during both stages, pre- and post-CD4 attachment may occur. Thus, to investigate the pre-attachment activity more precisely, we assessed the potential of bnAbs to neutralize when solely present before CD4 binding has been completed (Fig 7A). To this end, we first pre-incubated virus and bnAbs to allow for bnAb binding to the native spike. Concentrations of bnAbs were chosen to yield maximal inhibition (S5 Table). During subsequent spinoculation of the inoculum onto the target cells, virus binding to CD4 was initiated [25] allowing bnAb binding to epitopes exposed in the CD4-bound stage. Spinoculation was performed at room temperature to permit trimer binding to CD4 while arresting further structural rearrangements and fusion. The excess bnAbs and unbound virus were washed off and the infected target cells cultivated in absence of bnAbs. Efficacy in blocking virus entry was compared to controls in which bnAbs remained in the culture after spinoculation and therefore provided reference values for total neutralization activity covering bnAb action during the entire infection period (Fig 7A).

Pre-attachment neutralization efficacy monitored in our assay set-up provides a composite information on the capacity of an antibody to interact with native trimers on free virus and Env spikes that have already bound to CD4 but have not fully undergone Env rearrangements. Activity during these steps can be steered by several factors: i) the capacity of bnAbs to bind the native trimer with high affinity [72], ii) their efficacy to establish irreversible binding and neutralization (which is not lost upon washout of the bnAb) [69], iii) their capacity to block attachment to CD4 or an early stage of CD4 engagement, and iv) the capacity of the bnAb to induce a functional decay of the Env trimer [69].

Overall, bnAbs proved highly competent in neutralizing HIV during the pre-attachment phase. Low pre-attachment activity was predominantly observed with the subtype C strains ZM53, ZM109 and ZM214, particularly for bnAb PGT121, the V1V2- and the MPER-directed bnAbs and surprisingly with ZM214 also for T-20 (Fig 7B). CD4bs-directed bnAbs had the highest capacity to neutralize prior to CD4 attachment. Loss of CD4bs bnAb activity upon antibody removal was only observed with subtype C virus strains and to a relatively low extent (up to 25%). In general, high potencies of bnAbs against free virus transmission were linked with high pre-attachment activities while post-attachment activities (Fig 6C) and pre-attachment activities proved to be inversely linked (Fig 7C and S8 Fig), highlighting the importance of the capacity to bind the virus pre-CD4 engagement for bnAb efficacy against free virus spread. Of note, inhibitory concentrations for free virus and cell-cell transmission showed a high correlation, indicating that key aspects of the bnAb interaction with the viral Env during both entry processes must nevertheless be shared. Interestingly, the loss in neutralization activity during cell-cell transmission correlated with the pre-attachment activity, highlighting that bnAbs which preferentially act before CD4 engagement are less potent during cell-cell transmission confirming earlier findings [25]. In sum, our analyses suggest that the superior activity of bnAbs against free virus transmission is at least partially steered by their higher potential to access the virus prior to CD4 engagement. This feature seems to be less important for the efficacy in cell-cell neutralization as also highlighted by the activity of MPER-targeting bnAbs and T-20 during cell-cell transmission.

### Unique properties of V1V2- directed bnAbs CAP256-VRC26.08 and CAP256-VRC26.09

**Fig 8:**
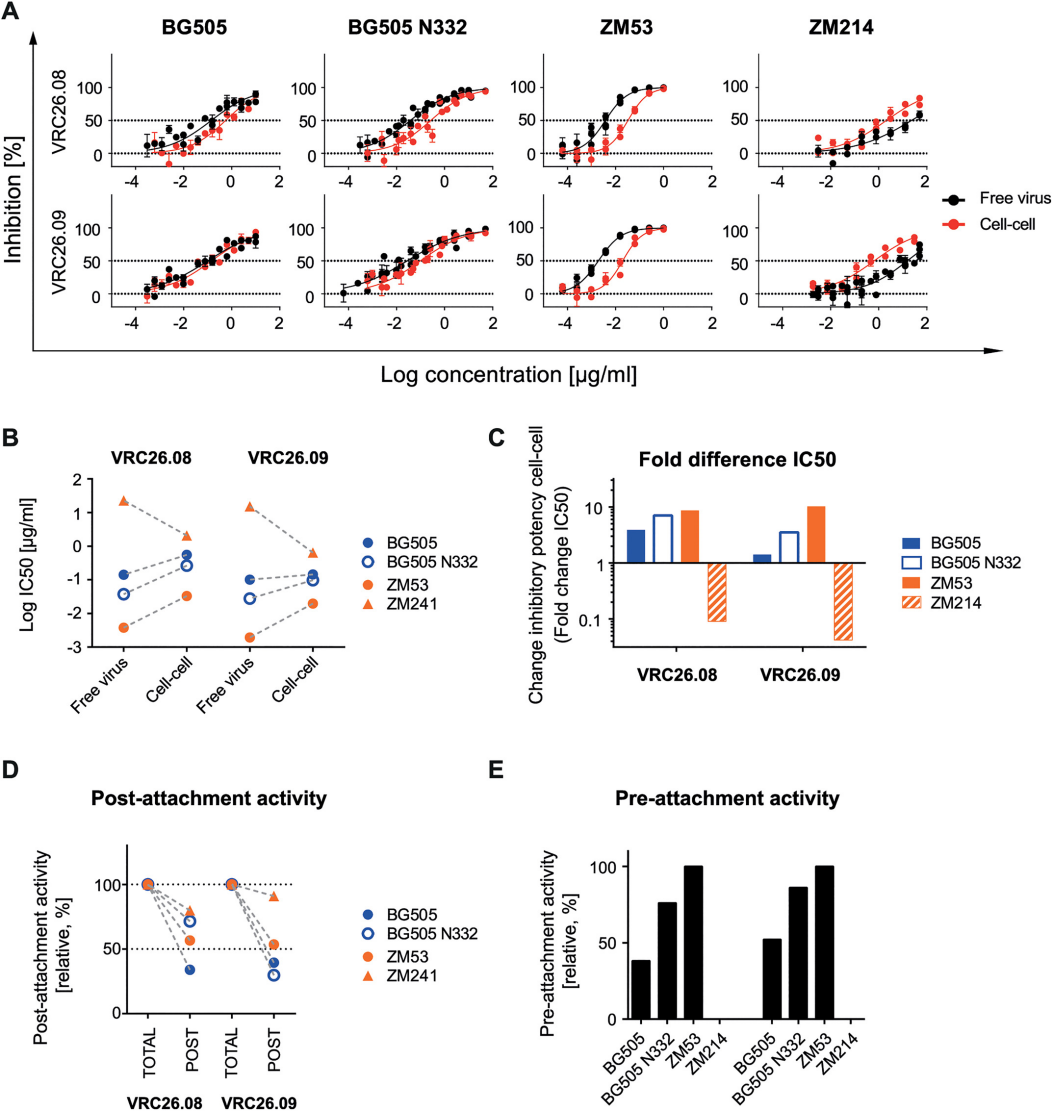
Unique properties of bnAbs CAP256-VRC26.08 and CAP256-VRC26.09. **A: Comparable potency of bnAbs CAP256-VRC26.08 and CAP256-VRC26.09 during free virus and cell-cell neutralization.** Inhibition of free virus (black circles) and cell-cell (red circles) transmission of the indicated virus strains by bnAbs CAP256-VRC26.08 and CAP256-VRC26.09 is shown. The graphs depict means and standard error of means (SEM, error bars) of two to three independent experiments performed in duplicates and curve fits to sigmoid dose response curves (variable slope). **B-C: Comparison of inhibitory activity of CAP256-VRC26.08 and CAP256-VRC26.09**. **B:** Comparison of IC_50_ values of free virus and cell-cell inhibition determined in **(A)** Subtype A and C viruses are denoted in blue and orange, respectively. **C:** Comparison of the change in inhibitory activity in cell-cell transmission relative to free virus inhibition (fold change IC_50_). **D: Comparison of the capacity to neutralize post-CD4 receptor engagement.** Activity of bnAbs added after attachment of virions to A3.01-CCR5 target cells was assessed and compared to samples, where bnAbs were present before and after attachment (total activity, set to 100% inhibition). Post- attachment inhibition is displayed relative to the total inhibition activity. Subtype A and C virus are denoted in blue and orange, respectively. One representative of two independent experiments is shown. **E: Comparison of the capacity to neutralize pre-CD4 receptor engagement.** The relative activity of bnAbs in the pre-attachment phase compared to total activity (bnAbs present before and after attachment) was assessed. Data are means of two to three independent experiments.

The V1V2-directed bnAbs proved to be less active during the pre-CD4 attachment phase of the subtype C viruses ZM53 and ZM109. Larger antibody and virus panels will be needed to define if this decreased neutralization activity of V1V2 bnAbs is a common feature of subtype C viruses or of the respective bnAbs probed. To obtain further insight, we tested two additional, clonally related V1V2-directed bnAbs, CAP256-VRC26.08 and CAP256-VRC26.09, which were isolated from a subtype C superinfected donor [73,74]. The two bnAbs were not able to neutralize the subtype B viruses in our panel but inhibited strains BG505 and BG505 N332 (subtype A) as well as ZM53 and ZM214 (both subtype C). Quite strikingly, the CAP256 bnAbs retained their activity during cell-cell neutralization at high levels for BG505, BG505 N332 and ZM53 (1.4–3.9-fold, 3.5-7-fold and 8.7-10-fold over free virus IC_50_) (Fig 8A–8C). The most surprising results were obtained for strain ZM214, which was the most neutralization resistant virus during cell-cell transmission in our panel. CAP256-VRC26.08 and CAP256-VRC26.09 proved to be considerably more potent against ZM214 in cell-cell transmission than free virus transmission (11-fold and 24-fold, respectively). Of note, ZM214 was not sensitive to any of the other V1V2-directed bnAbs tested (S5J–S5L Fig). Interestingly, the only other case where we found a bnAb to have a clearly superior activity during cell-cell transmission was also observed with a V1V2-directed bnAb and a subtype C virus, namely PGT145 against ZM53 (S5J Fig). In accordance with earlier observations (Fig 3), the capacity of the CAP256 bnAbs to block cell-cell and free virus transmission at identical levels was not linked to a particularly high inhibitory potency against this strain. In fact, neutralization of ZM214 required the highest CAP256 bnAb concentrations of all four viruses tested (Fig 8B, S3 Table). The subtype C strain ZM53 was the virus strain with the lowest IC_50_ for free virus and cell-cell inhibition (Fig 8A and 8B). In line with the other V1V2 bnAbs, we observed relatively high levels of post-attachment inhibition activity, which was highest for ZM214 with 80% and 77% for CAP256-VRC26.08 and CAP256-VRC26.09, respectively (Fig 8D). For ZM53, the virus strain most sensitive to CAP256 bnAb free virus neutralization, also the highest pre-attachment activities were observed (Fig 8E). In contrast, neutralization of ZM214, the least sensitive of the four probed strains in free virus inhibition, was completely abolished during the pre-attachment phase, again indicating that pre-attachment activity is linked to high bnAb potency. In sum, CAP256-VRC26.08 and CAP256-VRC26.09 differed in their properties from the other probed V1V2-directed bnAbs, PGT145, PG9 and PG16, which all failed to neutralize ZM214 and lacked pre-attachment activity against ZM53.

## Discussion

The progress in the development and characterization of highly potent bnAbs in the recent years has brought new hope for their use in therapeutic settings and the development of vaccine regimens eliciting alike responses [1]. Considering the increasing wealth of identified bnAbs, it will be important to focus these developments on the most promising candidates. This affords extensive *in vitro* evaluation and efficacy testing in animal models, the classical rhesus macaque SHIV model and increasingly also in humanized mouse models [7–14]. While efficacy testing in animal models is indispensable before clinical use of bnAbs, these models can currently not cover all aspects of human HIV-1 infection. Setting aside the issues of differences in immune systems, disease patterns and virus loads in these models, an inherent limitation are the virus strains used in animal experiments, as only few strains are available for *in vivo* application, especially in the case of the rhesus macaque model.

It has been consistently observed that the *in vivo* efficacy of neutralizing antibodies is considerably lower than their activity *in vitro* [12,13,17–19]. Amongst other factors, the decrease in neutralization activity during cell-cell transmission has been discussed as contributing factor for reduced *in vivo* efficacy [24–27]. Since breadth of bnAbs has so far only been assessed by free virus neutralization *in vitro*, differential activity loss of bnAbs *in vivo* due to a reduced activity in cell-cell transmission may not be fully captured by efficacy testing in animal models due to the limited number of virus strains that can be applied. Hence, if the capacity to potently block cell-cell transmission proves important for *in vivo* efficacy, tailored *in vitro* analyses are needed to dissect which bnAbs have the required activity. We therefore focused our study on defining the characteristics of free virus and cell-cell neutralization by bnAbs *in vitro* to provide additional functional information aiding the future selection of bnAbs for therapeutic and vaccine development.

We concentrated our analysis on four criteria of neutralization activity: (i) We probed the capacity of bnAbs to inhibit HIV-1 cell-cell transmission at comparable breadth and potency as free virus spread, considering that bnAbs that preserve high activity during cell-cell transmission across divergent strains can be viewed as attractive leads for therapeutic use and vaccine development. (ii) We investigated the probability of bnAb resistance to occur during free virus and cell-cell transmission. (iii) To derive more insight into the mechanisms of neutralization and the potency in the two transmission modes, we investigated the capacity of bnAbs to neutralize post-CD4 engagement. This was prompted by our previous work [25], which indicated that the capacity of antibodies to access the viral envelope post-CD4 engagement may be beneficial for maintaining activity during cell-cell transmission. Considering that trimer binding to CD4 initiates the formation of the virological synapse [31], antibodies that neutralize post-CD4 engagement could indeed benefit from a longer time window of action. (iv) Likewise, the capacity of bnAbs to act prior to CD4 engagement may be decisive for their activity. Ideally, a potent neutralizing antibody can be envisaged to have a high on-rate and low off-rate in binding the native Env spike before receptor binding, resulting in virtually irreversibly Env binding, obstruction of receptor binding or viral decay leading to irreversible neutralization [69,72,75]. Reversibility of binding and thereby neutralization can occur if the antibody has a high off-rate in binding the viral Env protein [69,72,76]. Rapid clearance before opsonized virions regain their infectivity upon antibody detachment may thus be crucial when binding and neutralization are reversible and potentially could require higher antibody doses that ascertain optimal triggering of effector functions and clearance [21–23].

Due to the complexity of the assays, neutralization of cell-cell transmission has previously only been assessed for a relatively small number of antibodies and HIV-1 isolates [24–30]. An improved pseudovirus-based assay system to study cell-cell transmission allowed us to screen the cell-cell inhibition capacity of a wide range of virus-bnAb combinations. In sum, we probed the sensitivity of 11 virus strains from subtypes A, B and C to a panel of 16 bnAbs targeting the CD4bs, V3 glycan region, the V1V2 loop and the MPER domain during free virus and cell-cell transmission. In accordance with previous reports, we observed an overall decreased neutralization activity of bnAbs to inhibit cell-cell compared to free virus spread with few exceptions. Interestingly however, activities against both transmission routes varied substantially between the different virus strains tested and we could not identify a single bnAb that equally blocked free virus and cell-cell transmission over a broad range of HIV-1 strains. Many of the probed bnAbs performed in a similar range. Against the viruses investigated, the most potently and consistently neutralizing bnAbs for both pathways were the CD4bs-directed bnAb PGV04 and the MPER-directed bnAb 10E8. Amongst loop-specific bnAbs, which all had genuinely lower breadth, PGT121 was the most effective in combining breadth, potency and a low loss during cell-cell transmission.

Intriguingly, while inhibitory concentrations required for free virus and cell-cell inhibition correlated, a high potency against free virus spread did not ensure a lower loss in activity during cell-cell transmission (Figs 3, 7C and S8 Fig). On the contrary, we identified a negative correlation between the inhibitory concentrations required for blocking free virus spread (IC_50_) and the loss in activity during cell-cell transmission (fold change of the corresponding IC_50_ values). We frequently observed that bnAbs that already required high concentrations to neutralize free virus spread maintained similar neutralization activities against cell-cell transmission. Collectively, this suggests that properties that render a bnAb highly potent in neutralizing free virus spread are not equally relevant for inhibiting cell-cell transmission. It has been recently suggested that virus particles in free virus and cell-cell transmission may differ in their maturation status. While free virus might be largely matured, cell-cell transmitted virus may still be in an immature form which potentially could also change the trimer conformation through differential gp41 cytoplasmic tail Gag interactions, also affecting antibody binding [49]. Additionally, immediately upon virus release, virions may carry more intact trimers which rapidly decay over time, adding to the intrinsic differences in trimer content observed across isolates [77].

A factor that likely steers free virus neutralization is the on-rate of the antibody for binding the native trimer before receptor engagement. If the epitope is optimally exposed on the free virus, the antibody can bind rapidly, with high affinity (and ideally irreversibly), resulting in quick and potent neutralization of the free virus. If firm bnAb binding to the native trimer requires substantial conformational rearrangements (e.g. induced by the bnAb binding itself or CD4- and co-receptor engagement), antibody binding and neutralization kinetics of free virus transmission will be slower and likely less efficient. Hence in the latter case, neutralization of free virus and cell-cell transmission may both occur predominantly after trimer binding to CD4 [31]. In summary, we observed that despite their inherent high activity against free viruses, bnAbs can substantially differ in their capacity to block cell-cell transmission, highlighting that the potency in inhibiting free virus spread does not allow to reliably draw conclusions on the neutralization performance during cell-cell transmission. In our study, decreased sensitivity to neutralization during cell-cell transmission proved not to be a characteristic of specific viruses or bnAbs, confirming the need to include a range of viruses when assessing bnAb activity during cell-cell transmission. This was probably best evidenced by the divergent patterns we observed for the three subtype C viruses in our panel. While inhibition of ZM53 and ZM109 across the probed bnAbs required comparatively low IC_50_ for free virus inhibition, paired with relatively modest activity losses during cell-cell transmission, we observed large differences in capacity for free virus and cell-cell neutralization of ZM214.

The only Env-directed inhibitor that we identified to exert a widely preserved activity in both transmission modes was the fusion inhibitor T-20 [78–80]. Strikingly, while neutralization capacities of bnAbs in free virus and cell-cell transmission varied substantially, the gp41-directed inhibitor T-20 retained its potency in both transmission modes across viruses at comparative levels. Defining the precise properties of T-20 that preserve its activity during cell-cell transmission in future studies will hence be of interest as this may provide guidance on what mechanistic features are required for high efficacy in cell-cell neutralization and potentially also *in vivo* activity. Of note, as T-20 targets the six-helix bundle formation, a step in virus entry post-receptor engagement, kinetics of T-20 inhibition could indeed be similar for free virus and cell-cell transmission. While T-20 (4.5 kDa) is considerably smaller than antibodies (150kDa), its size alone may not be decisive for its activity during cell-cell transmission. In our previous studies, we demonstrated that both, CD4-directed neutralizing antibodies and the CD4-peptide mimetic CDM47 (2.9 kDa; [81]) equally loose potency during cell-cell inhibition [25].

**Fig 9:**
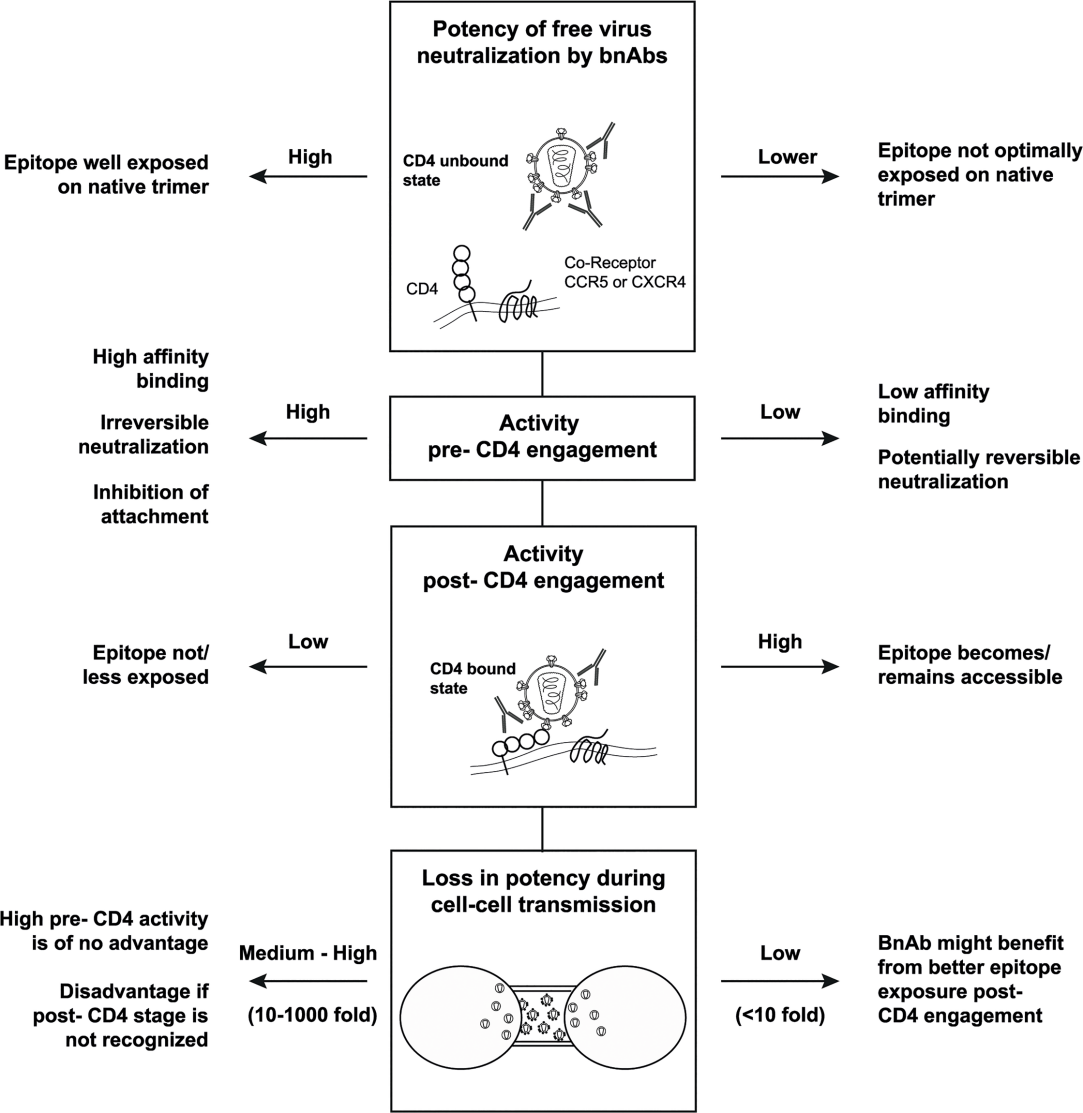
Interplay of bnAb features that shape neutralization potency. Differential features shape the neutralization activity of bnAbs. Two main reactivity patterns can be observed: (i) BnAbs with high potency against free virus spread and high pre-attachment activity. (ii) BnAbs with lower efficacy against free virus spread, decreased pre-attachment but high post-CD4 attachment activity and retained activity during cell-cell transmission.

With few exceptions, neutralization of free virus by CD4bs- and V3-directed bnAbs proved to be highly potent during the pre-attachment phase while neutralization by V1V2- and MPER-targeting bnAbs depended also on post-attachment activity for several virus strains. A decreased efficacy during the pre-attachment phase could be caused by a failure of the bnAbs to bind the native trimer, to interfere with attachment to CD4 or to irreversibly neutralize the virus. If virus neutralization remains reversible for an extended time period, this could potentially cause a reduced *in vivo* efficacy if opsonized viral particles are not rapidly cleared [21–23], highlighting the need to further evaluate how reversibility of neutralization affects the *in vivo* efficacy of bnAbs. Interestingly, we found that high potency against free virus was linked with high pre-attachment activity (Fig 7C and S8 Fig) while low pre-attachment activity was linked with activity post-CD4 attachment and lower losses during cell-cell transmission. Although it is not possible to generalize our findings for all antibody-virus combinations studied, the reactivity patterns we observed tended to fall into two distinct groups (Fig 9): BnAbs with high potency against free virus spread show a high activity against the virus prior to CD4-binding, indicating optimal access of the epitope on the native trimer and high affinity binding. BnAbs with lower neutralization activity against free virus spread lack pre-attachment activity, show potent neutralization post-CD4 attachment and retain activity during cell-cell transmission, indicating suboptimal binding to the native trimer and improved access to the epitope post-CD4 engagement.

To date, the relative impact of free virus and cell-cell spread *in vivo* has not been determined. If cell-cell transmission should prove to contribute to viral spread *in vivo*, the decreased sensitivity of this transmission mode to antibody neutralization may indeed be of concern as it may provide viruses with a possibility to better tolerate antibody pressure and acquire resistance mutations. Here, we investigated if the chances that resistance mutations occur indeed differ for free virus and cell-cell transmission. As our mathematical analysis revealed, cell-cell transmission proved to be substantially more prone to give rise to escape mutants than free virus transmission. This highlights the importance of controlling virus replication via the cell-cell transmission pathway even if the contribution of this transmission mode should proof to occur to a lesser extent than free virus spread in infected individuals. The selection of bnAbs that are developed further for clinical use need to be carefully designed to ascertain *in vivo* efficacy to the best of our knowledge. Most likely, the increasing data on currently ongoing bnAb efficiency in *in vivo* testing will give us an improved picture on which features determine the *in vivo* efficacy of bnAbs. These will probably not only involve potency and breadth against free viruses but also favorable pharmacokinetics of bnAbs [11,26] and their capacity to elicit effector functions and to induce rapid virus clearance [21–23]. As highlighted by our analyses, functional characteristics such as the efficacy to neutralize virus during cell-cell transmission and the different stages of the entry process are important features that shape the antibodies’ activity, steer their potency and their potential for mutant selection. Hence, these characteristics should be considered in future when evaluating bnAbs for clinical use. The high variability of bnAb activity across genetically divergent viruses and their varying capacities to block both transmission modes and to differentially neutralize pre- and post CD4 attachment further highlights the need to create powerful combination of bnAbs, either by multi-component vaccines or antibody cocktails [82] in passive immunization to ensure that all mechanistic features required for effective virus control are represented.

## Materials and Methods

### Ethics statement

Peripheral blood mononuclear cells (PBMC) were purified from buffy coats from anonymous blood donations from healthy individuals obtained by the Zurich Blood Transfusion Service (http://www.zhbsd.ch/) under a protocol approved by the local ethics committee.

### Reagents

Properties and sources of antibodies and inhibitors used in this study are listed in S2 Table. We thank D. Burton, J. Mascola, M. Nussenzweig, M. Connors, P. Moore and L. Morris for providing antibodies and antibody expression plasmids for this study either directly or via the NIH AIDS Research and Reference Reagent Program (NIH ARP).

### Cells

293-T cells were obtained from the American Type Culture Collection (ATCC), TZM-bl [16] were obtained from the NIH ARP. A3.01-CCR5 cells were described previously [25,83]. 293-T and TZM-bl cells were cultivated in DMEM with 10% heat inactivated FCS and 1% Penicillin/Streptomycin. A3.01-CCR5 cells were maintained in RPMI with 10% heat inactivated FCS and 1% Penicillin/Streptomycin.

Stimulated PBMC were isolated from healthy donors as described [61] and cultivated in RPMI with 10% heat inactivated FCS, 1% Penicillin/Streptomycin and 100 units/ml (U) IL-2.

### Viruses

Properties and sources of plasmids encoding the envelope of strains BG505 (subtype A), JR-FL, JR-CSF, SF162, DH123, PVO.4, REJO, THRO (all subtype B), ZM214, ZM109, ZM53 (all subtype C) are listed in S1 Table.

The T332N envelope point mutation in BG505 was generated by site-directed mutagenesis (Agilent QuikChange II XL) according to the manufacturer instructions. The point mutant envelope (denoted as BG505 N332) was sequenced by in-house Sanger sequencing to confirm presence of the desired mutations and absence of unintended sequence changes.

For the production of single-round replicating pseudovirus stocks, 293-T cells were transfected with the respective viral backbones and *env* expression plasmids as described [61]. The following backbone constructs were used: The luciferase reporter HIV-1 pseudotyped vector pNLlucAM [84] and the NL4-3 based pseudotyped vector with integrated inGluc reporter construct (NLinGluc [56]; a gift from Dr. M. Johnson).

Infectivity of reporter viruses was quantified by titration of virus containing supernatants on 1*10^4^ TZM-bl or 5*10^4^ A3.01-CCR5 in a 1:3 ratio starting from 100 µl virus solution/well. Infection of target cells was assessed by either measuring the Gaussia luciferase signal from the supernatant using the Renilla Luciferase Assay System (Promega, Madison Wisconsin, USA) for NLinGluc reporter viruses or by measuring the firefly luciferase activity from the lysed cells using firefly luciferase substrate (Promega, Madison Wisconsin, USA) for NLlucAM reporter viruses.

Replication competent virus subtype B isolates JR-FL and JR-CSF were propagated on CD8 T-cell-depleted PBMC and titered as described [85].

### Neutralization of free Env-pseudotyped virus on A3.01-CCR5 and primary T-cells

Free virus inhibition by bnAbs and inhibitors was assessed on A3.01-CCR5 cells using Env-pseudotyped NLlucAM reporter viruses in all experiments except Fig 1A, where Env-pseudotyped NLinGluc virus was used. Virus input was chosen to yield virus infectivity corresponding to a firefly activity of around 10’000 relative light units (RLU) per 96 well in absence of inhibitors. Viruses and indicated doses of inhibitors were pre-incubated for 1 h at 37°C and added to 5*10^4^ A3.01-CCR5 target cells per 96 well in the presence of 10 µg/ml diethylaminoethyl (DEAE, Amersham Biosciences, Connecticut, USA). For free virus neutralization assays on primary T cells (PBMC), virus-inhibitor mix was added to 1.5*10^5^ PBMC per 96 well in the presence of 8 µg/ml Polybrene. After 65 h incubation at 37°C, infection was assessed by luciferase production after cell lysis and addition of firefly luciferase substrate (Promega, Madison, Wisconsin, USA). Emitted RLU were quantified on a Dynex MLX luminometer (Dynex Technologies Inc., Chantilly, Virginia, USA). The inhibitor concentrations causing 50% reduction in viral infectivity (50% inhibitory concentration; IC_50_) were calculated by fitting pooled data from two to three independent experiments to sigmoid dose response curves (variable slope) using GraphPad Prism. If 50% inhibition was not achieved at the highest or lowest inhibitor concentration, a greater-than value was recorded. For comparison, neutralization assays were also performed with NLinGluc reporter viruses (Fig 1D, S2 Fig) with the same experimental setup. Infectivity was assessed by measuring Gaussia luciferase activity in the culture supernatant.

### Assessing neutralization activity during cell-cell transmission of 293-T to A3.01-CCR5 cells

Neutralization of HIV-1 cell-cell transmission was analyzed using NLinGluc virus transfected 293-T cells donor and A3.01-CCR5 target cells in all experiments, except in Fig 1F and S4 Fig, where also PBMC-PBMC transmission was used. The cell-cell transmission assay utilizes an NL4-3 derived pseudotyped HIV backbone carrying an intron-regulated Gaussia luciferase LTR-reporter construct termed inGluc which is co-transfected with an *env* plasmid into donor cells [41,55,56]. The intron and a reverse orientation of the reporter gene prohibit the luciferase expression in the transfected cell. Production of functional Gaussia luciferase requires correct splicing, packaging into viral particles and infection of and expression in the target cells. This allows for a clear separation of cell-cell transmission and cell fusion events [41,55].

To exclusively study cell-cell transmission, free virus infectivity was restricted by the omission of DEAE in the infection media as previously described [25].

For assessing neutralization of cell-cell transmission, 293-T cells were transfected with *env* and NLinGluc plasmids in a 1:3 ratio. 6 h post transfection, 5*10^3^ transfected cells were seeded in 50 µl per 96 well and serial dilutions of inhibitors in 50 µl per 96 well were added. After 1 h incubation at 37°C, 1.5*10^4^ A3.01-CCR5 target cells in 100 µl RPMI medium were added to the 293-T–inhibitor mix per 96 well. After 65 h of incubation at 37°C, Gaussia luciferase activity in the supernatant was quantified using the Renilla Luciferase Assay System (Promega, Madison Wisconsin, USA) according to the manufacturer’s instructions. Neutralization data were analysed with GraphPad Prism as described above.

### Neutralization of HIV-1 cell-cell transmission in primary T cells

As DEAE omission cannot be used for restricting free virus infectivity in PBMC, rhTRIM5α restriction in the primary target cells was used, as recently described [25].

Briefly, PBMC were transduced with a bicistronic lentiviral GFP and rhTRIM5α expression vector ([86]; provided by J.L.Riley) one day after isolation and stimulation with OKT3 and 2 µg/ml CD28 in RPMI with 8 µg/ml Polybrene. Four days after transduction, rhTRIM5α positive cells were detected via the bicistronic GFP expression by FACS and sorted using a FACS AriaIII (BD Biosciences, New Jersey, USA).

To analyze the neutralization capacity of inhibitors, stimulated, CD8-depleted PBMC were infected with replication competent virus stocks for five days at 37°C and a MOI around 0.01. PBMC were washed twice to remove free virions and 1*10^4^ PBMC in 50 µl RPMI with 100 U IL-2 per 96 well were pre-incubated with serial dilutions of inhibitors for 1 h at 37°C. For co-culture, 1*10^4^ rhTRIM5α transduced PBMC in 100 µl RPMI with 100 U IL-2 per 96 well were added to the donor cell- inhibitor mix. After three days of co-culture at 37°C, infectivity was assessed by intracellular p24 staining, analyzed with a FACS CyAn ADP (Beckman Coulter). Neutralization data of infected, rhTRIM5α-positive cells were analyzed with FlowJo software (TreeStar, Oregon, USA) and GraphPad Prism as described.

### Assessing pre- and post-attachment activity

The inhibitory capacities of inhibitors at a pre- and post-attachment state of NLlucAM reporter viruses on A3.01-CCR5 target cells were analyzed as previously described [25].

In short, to test total inhibitory activity covering both, the pre- and post-attachment stage, NLlucAM reporter viruses yielding a firefly activity of around 10’000 relative light units (RLU) per 96 well in absence of inhibitors were pre-incubated with inhibitors for 1 h at 37°C. The pre-treated virus was then spinoculated onto 1*10^5^ A3.01-CCR5 target cells in RPMI with 50 µM Hepes and 10 µg/ml DEAE per 96 well for 2 h at 1200 g and 23°C. Unbound virus and inhibitors remained with the cells during the subsequent cultivation at 37°C. These conditions provide information of the cumulative activity of an inhibitor before and after attachment, for brevity we refer to it as total activity. To test inhibitory capacity at the post-attachment stage, NLlucAM reporter viruses were first spinoculated onto A3.01-CCR5 cells which were then incubated with inhibitors for 1 h at 23°C before raising the temperature to 37°C. All samples were incubated for 65 h at 37°C and infectivity was determined by firefly luciferase production from the lysed cells as described. Total activity samples were set to 100% inhibition and post-attachment inhibition was expressed relative to this value.

For assessing neutralization activity only at the pre-attachment step, following pre-treatment and spinoculation, unbound virus and inhibitors were washed off during two washing steps at 450 g for 2 min. Therefore, this condition provides information on how much of the binding and neutralization can occur before finalisation of CD4 engagement and if the affinity of the binding is high enough to sustain washing. The inhibitory capacities measured after washout of bnAbs reflect neutralization that was initiated prior to receptor engagement. Pre-attachment activity is expressed in relation to the total inhibitory activity which was set to 100%.

### Theoretical considerations for assessing the propensity to select for neutralization resistant mutants in cell-cell and free virus infection pathways

To compare the probabilities that a mutant variant arises via the free virus pathway versus the cell-cell pathway for a certain antibody concentration, we first derive an analytical expression for these probabilities, *p*_*M*_(*c*, *type*), where *c* is the antibody concentration and *type* is either cell-cell, *cc*, or free- virus, *fv*.

This probability is the product of the probability that a mutation *M* arises given cell infection, *I*, and the probability that infection happens:

$$p_{M}(c,type)=P(M|I,c,type)\times P(I,c,type)=P(M|I,c,type)\times P(I|c,type)\times P(c,type)$$

The last factor is one. The probability that a mutant arises, given a cell is infected via the pathway *type*, *P*(*M* | *I*,*c*,*type*), does not depend on the concentration of antibodies, *c* as all mutations arise during reverse transcription in the infected cell in a process that is not affected by the antibodies.

The probability that a mutant arises, given infection in the cell-cell pathway, is denoted *P*(*M* | *I*,*c*,*cc*) = *μ*_*cc*_ and the same probability for the free virus pathway *P*(*M* | *I*,*c*,*fv*) = *μ*_*fv*_.

Despite the fact that point mutations occurring via reverse transcription should happen with the same frequency independent of the two pathways, *μ*_*cc*_ and *μ*_*fv*_ can differ due to recombination. Recombination is more likely to happen, when a cell is infected with more than one virion, which might be more frequent in cells transfected via the cell-cell route [37,38,46]

To include the possibility of differing point mutation rates, let *μ*_*cc*_ = *δμ*_*fv*_, with *δ* > 0. If *δ* ranges between 0 and 1, the probability that a mutant arises in a cell that was infected via the cell-cell pathway is lower than that for a free virus infected cell. If *δ* > 1, the probability that a mutant arises via the cell-cell pathway is higher than for the free virus pathway. The latter parameterization is biologically more likely due to the reasons mentioned above.

The probability that a cell becomes infected given a certain antibody concentration, *c*, is:

P(I|c,type)=π(1−%inhib(c,type)/100)

*π* is the probability of cell infection without any antibody and %*inhib*(*c*, *type*) is the inhibition determined in *in vitro* infection experiments,

$$\%inhib(c,type)=i_{max}\frac{c^{m}}{c^{m}+IC\;50^{m}}$$

By dividing the probability that a mutation arises via the cell-cell pathway by that via free virus infection we obtain:

$$\gamma (c)=\frac{p_{M}(c,cc)}{p_{M}(c,fv)}=\frac{\delta (1-i_{\text{max},cc}c^{m_{cc}}\;{\left(c^{m_{cc}}+IC50_{cc}^{m_{cc}}\right)}^{-1})}{1-i_{\text{max},fv}c^{m_{fv}}\;{\left(c^{m_{fv}}+IC50_{fv}^{m_{fv}}\right)}^{-1}}$$

For this analysis, we used the IC_50_ and slope values (S3 and S4 Tables) obtained by inhibition data analysis performed with GraphPad Prism. The formula are implemented in the R statistical analysis software [87].

### Statistical analysis

Statistical analyses (correlation analyses according to Spearman using the untransformed data sets) were performed using GraphPad Prism.

## Supporting Information

S1 Table Overview and characteristics of the virus strains used. (DOCX)

S2 Table Origin and specificities of bnAbs and inhibitors. (DOCX)

S3 Table Overview of inhibitory capacities. IC_50_ of free virus and cell-cell inhibition and fold changes IC_50_ between the two transmission modes for all sensitive bnAb-virus combinations. Where IC_50_ for cell-cell transmission could not be determined in the probed concentration range, the highest bnAb-concentration tested is indicated (bold letters). For the calculation of the fold change IC_50_, the IC_50_ of insensitive bnAb-virus combinations was nominally set to a value of two times the highest ineffective bnAb-concentration tested. These values are indicated in bold letters.
(DOCX)

S4 Table Overview of slope values. Slope values, m, were determined from free virus and cell-cell inhibition assays for all bnAb-virus combinations (S5A–S5P Fig) by fitting the Hill curve equation c^m^/(c^m^ +IC_50_^m^) to the inhibition data. Parameters are shown for all bnAb-virus combinations for which this fitting procedure was successful.
(DOCX)

S5 Table Concentrations used and maximal neutralization obtained in the assessment of pre- and post-attachment activity of bnAbs. Maximal concentrations used for the assays determining pre- and post-attachment and total activity (Figs 6, 7 and 8) and maximal percentage of neutralization obtained are indicated.
(DOCX)

S1 Fig DEAE dependency of free HIV-1 viruses. DEAE dependency of a range of Env-pseudotyped NLlucAM reporter viruses is shown. Free Env NLlucAM pseudoviruses were titrated on TZM-bl cells in 96-well plates in presence (black circles) or absence (green squares) of 10 µg/ml diethylaminoethyl (DEAE). The graphs show means and standard error of means (SEM, error bars) of two to three independent experiments performed in duplicates and curve fits to sigmoid dose response curves (variable slope). Net charges of the V3 region were calculated with the Innovagen AB peptide property calculator (http://pepcalc.com/ppc.php) and are indicated with bold numbers.
(EPS)

S2 Fig NLinGluc (Gaussia) and NLlucAM (firefly) luciferase reporter viruses yield comparable results in free virus inhibition assays. Free virus inhibition of JR-FL NLinGluc (yellow circles) and JR-FL NLlucAM (black circles) reporter virus by the indicated bnAbs was compared. For both viruses, 100% infectivity was determined in cultures without inhibitor. The graphs show means and standard error of means (SEM, error bars) of two to three independent experiments performed in duplicates and curve fits to sigmoid dose response curves (variable slope).
(EPS)

S3 Fig Equal neutralization sensitivity of free virus infection of A3.01 CCR5 and PBMC target cells. **A-E:** Inhibition of free virus infection of PBMC (blue squares) or A3.01 CCR5 (black circles) by different bnAbs was studied using Env-pseudotyped NLlucAM reporter viruses JR-FL **(A)**, JR-CSF **(B)**, SF162 **(C)**, DH123 **(D)** and ZM53 **(E)** NLlucAM. For both cell types, 100% infectivity was determined in cultures without inhibitor. The graphs show means and standard error of means (SEM, error bars) of two to three independent experiments performed in duplicates and curve fits to sigmoid dose response curves (variable slope).
(EPS)

S4 Fig Equal sensitivity to neutralization in 293-T-A3.01 CCR5 and PBMC-PBMC transmission. **A-B:** The capacity of the indicated bnAbs to block cell-cell transmission was assessed in co-cultures of JR-FL **(A)** and JR-CSF **(B)** NLinGluc-transfected 293-T with A3.01-CCR5 (black circles) and JR-FL and JR-CSF infected PBMC with rhTRIM5α-transduced PBMC (blue squares). Infectivity was assessed via determination of Gaussia luciferase activity in the 293-T-A3.01 co-cultures or via intracellular p24 staining and flow cytometry analysis for the PBMC co-cultures. For both co-cultures, 100% infectivity was determined in cultures without inhibitor. The graphs show means and standard error of means (SEM, error bars) of two to three independent experiments and curve fits to sigmoid dose response curves (variable slope).
(EPS)

S5 Fig Free virus and cell-cell inhibition by bnAbs. **A-P:** Addendum to Fig 2. Inhibition of free virus (black circles) and cell-cell (red circles) transmission of subtype A, B and C virus strains by the indicated bnAbs was studied. The graphs show means and standard error of means (SEM, error bars) of two to three independent experiments performed in duplicates and curve fits to sigmoid dose response curves (variable slope). Subfigures A-P show free virus and cell-cell inhibition profiles determined for each bnAb.
(PDF)

S6 Fig Neutralization capacities of free virus and cell-cell inhibition are virus strain- and bnAb epitope-dependent. **A:** Comparison of free virus (black bars) and cell-cell (red bars) IC_50_ for all sensitive bnAbs-virus pairs probed. Lines of the box and whisker-plots indicate the first quartile, the median and the third quartile (from top to bottom). Whiskers show the minimum to maximum values recorded. **B:** Comparison of fold change IC_50_ of cell-cell transmission compared to free virus infection analysed by bnAb class and virus subtypes. Subtype A, B and C viruses are denoted in blue, black and orange, respectively. All subtypes combined are displayed in grey. Lines of the box and whisker-plots indicate the first quartile, the median and the third quartile (from top to bottom). Whiskers show the minimum to maximum values recorded. **C:** Comparison of fold change IC_50_ of cell-cell transmission compared to free virus infection analysed by virus strain. Lines of the box and whisker-plots indicate the first quartile, the median and the third quartile (from top to bottom). Whiskers show the minimum to maximum values recorded.
(EPS)

S7 Fig Mutant occurrence probability in case that the per site mutation rate is five times higher in cell-cell transmission. The panels show how much more likely it is that an escape variant arose via the cell-cell pathway than during free virus spread. The light grey areas correspond to the concentrations for which an escape variant is more likely to be formed during cell-cell spread. The dark grey areas show a predominant formation during free virus spread. In Fig 5 we calculated these ratios with equal per site mutation rates independent of the route of transmission. Due to higher virus copy numbers in infected cells via the cell-cell in comparison to the free virus pathway, recombination could be more abundant in cells infected via the cell-cell pathway. This would result in a higher per site mutation rate (Materials and Methods) for the cell-cell pathway. Here we show the ratio of the mutant occurrence probabilities in case the per site mutation rate is five times higher in cells infected via cell-cell transmission than via free virus transmission.
(EPS)

S8 Fig Pre-attachment activity correlates with high potency against free virus, decreased post-attachment inhibition capacity and loss in activity during cell-cell transmission. Interdependencies of pre-attachment, post-attachment inhibition, IC_50_ for free virus inhibition, IC_50_ for cell-cell inhibition and loss in activity during cell-cell inhibition. Correlations were determined by Spearman correlation based on the untransformed data sets. R and p values are depicted. n.s. indicates a non-significant correlation. Direct correlation is denoted in blue, inverse correlation in orange. Subtype A, B and C viruses are denoted in blue, black and orange, respectively.
(EPS)
