# Supplementary material for: Capacity of Broadly Neutralizing Antibodies to Inhibit HIV-1 Cell-Cell Transmission Is Strain- and Epitope-Dependent
Source: PLoS Pathog. 2015 Jul 9;11(7):e1004966. doi: 10.1371/journal.ppat.1004966 (PMC4497647; doi:10.1371/journal.ppat.1004966)
Supplement: S1 Table — (DOCX) [file ppat.1004966.s001.docx]

| **Envelope** | **Subtype** | **Co-receptor** | **Sensitivity** | **Disease stage** | **V3 net charge^1^** | **Source** |
| --- | --- | --- | --- | --- | --- | --- |
| JRFL | B | CCR5 | Tier 2 | Chronic | 3.2 | Koyanagi et al. 1987  Science. 236(4803):819-22. |
| JRCSF | B | CCR5 | Tier 2 | Chronic | 5.2 | Koyanagi et al. 1987  Science. 236(4803):819-22. |
| SF162 | B | CCR5 | Tier 1A | Chronic | 3.1 | Cheng-Mayer et al. 1988  Ann Neurol.23 S58-61. |
| DH123 | B | CCR5 and CXCR4 | Tier 1 | Chronic | 4.1 | Shibata et al. 1995  J.Virol. 69 4453-4462. |
| PVO.4 | B | CCR5 | Tier 3 | Acute | 3 | Li et al. 2005  J.Virol.79 10108-10125 |
| REJO  c/2864 | B | CCR5 | Tier 2 | T/F | 4.1 | Li et al. 2005  J.Virol.79 10108-10125 |
| THRO  c/2626 | B | CCR5 | Tier 2 | T/F | 4.1 | Li et al. 2005  J.Virol.79 10108-10125 |
| BG505 | A | CCR5 | Tier 3 | T/F | 3.1 | Wu et al. 2006  J.Virol.80 835-844 |
| ZM53  M.PB12 | C | CCR5 | Tier 2 | Acute | 4 | Derdeyn et al. 2004 Science.303 2019-2022 |
| ZM109  F.PB4 | C | CCR5 | Tier 1B | Acute | 4 | Derdeyn et al. 2004 Science.303 2019-2022 |
| ZM214  M.PL15 | C | CCR5 | Tier 2 | Acute | 3.1 | Derdeyn et al. 2004 Science.303 2019-2022 |
