# Supplementary material for: Capacity of Broadly Neutralizing Antibodies to Inhibit HIV-1 Cell-Cell Transmission Is Strain- and Epitope-Dependent
Source: PLoS Pathog. 2015 Jul 9;11(7):e1004966. doi: 10.1371/journal.ppat.1004966 (PMC4497647; doi:10.1371/journal.ppat.1004966)
Supplement: S2 Table — (DOCX) [file ppat.1004966.s002.docx]

| **Inhibitor** | **Drug class** | **Target/Epitope** | **Reference** | **Source** |
| --- | --- | --- | --- | --- |
| **b12** | Antibody | gp120, CD4-bs | Barbas et al. 1992 PNAS.89(19):9339-43 | D. Burton, The Scripps Research Institute, La Jolla, USA |
| **VRC01** | Antibody | gp120, CD4-bs | Wu, Yang et al. 2010 Science.329(5993):856-61 | J. Mascola, Vaccine Research Center, National Institute of Health, Maryland, USA |
| **PGV04** | Antibody | gp120, CD4-bs | Wu, Yang et al. 2010 Science.329(5993):856-61 | J. Mascola, Vaccine Research Center, National Institute of Health, Maryland, USA |
| **3BNC117** | Antibody | gp120, CD4-bs | Scheid et al. 2011 Science 16;333(6049):1633-7 | NIH AIDS Reagent Program, Division of AIDS, NIAID,  M. Nussenzweig, The Rockefeller University, New York, USA |
| **NIH45-46** | Antibody | gp120, CD4-bs | Scheid et al. 2011 Science 16;333(6049):1633-7 | NIH AIDS Reagent Program, Division of AIDS, NIAID,  M. Nussenzweig, The Rockefeller University, New York, USA |
|  |  |  |  |  |
| **PG9** | Antibody | gp120, V1V2 peptidoglycan | Walker et al. 2009 Science 326(5950):285-9 | D. Burton, The Scripps Research Institute, La Jolla, USA |
| **PG16** | Antibody | gp120, V1V2 peptidoglycan | Walker et al. 2009 Science 326(5950):285-9 | D. Burton, The Scripps Research Institute, La Jolla, USA |
| **PGT145** | Antibody | gp120, V1V2 peptidoglycan | Walker et al. 2011 Nature 477(7365):466-70 | D. Burton, The Scripps Research Institute, La Jolla, USA |
| **2G12** | Antibody | Outer domain glycan | Trkola et al.1996 J Virol.70(2):1100-8 | D. Katinger, Polymun, Vienna, Austria |
|  |  |  |  |  |
| **PGT121** | Antibody | gp120, V3 peptidoglycan | Walker et al. 2011 Nature 477(7365):466-70 | D. Burton, The Scripps Research Institute, La Jolla, USA |
| **PGT125** | Antibody | gp120, V3 peptidoglycan | Walker et al. 2011 Nature 477(7365):466-70 | D. Burton, The Scripps Research Institute, La Jolla, USA |
| **PGT128** | Antibody | gp120, V3 peptidoglycan | Walker et al. 2011 Nature 477(7365):466-70 | D. Burton, The Scripps Research Institute, La Jolla, USA |
| **PGT135** | Antibody | gp120, V3 peptidoglycan | Walker et al. 2011 Nature 477(7365):466-70 | D. Burton, The Scripps Research Institute, La Jolla, USA |
|  |  |  |  | D. Burton, The Scripps Research Institute, La Jolla, USA |
| **2F5** | Antibody | gp41, MPER (671-676) | Muster et al.1993 J Virol.67(11):6642-7 | D. Katinger, Polymun, Vienna, Austria |
| **4E10** | Antibody | gp41, MPER (662-667) | Stiegler et al. 2001 AIDS Res Hum Retroviruses. 10;17(18):1757-65 | D. Katinger, Polymun, Vienna, Austria |
| **10E8** | Antibody | gp41, MPER | Huang et al. 2012 Nature 491(7424):406-12 | NIH AIDS Reagent Program, Division of AIDS, NIAID |
|  |  |  |  |  |
| **CAP256 VRC26.08** | Antibody | V1V2 | Doria-Rose et al. 2014, Nature 509, 55–62 | L. Morris, P. Moore, National Institute for Communicable Diseases (NICD), South Africa |
| **CAP256 VRC26.09** | Antibody | V1V2 | Doria-Rose et al. 2014, Nature 509, 55–62 | L. Morris, P. Moore, National Institute for Communicable Diseases (NICD), South Africa |
|  |  |  |  |  |
| **T20 (Fuzeon)** | Peptide, fusion inhibitor | gp41, Fusion peptide | Wild et al.1993 AIDS Res Hum Retroviruses. 9(11):1051-3 | Purchased from Roche Pharmaceuticals |
| **Atazanavir Sulfate** | Azapeptide, Protease inhibitor | Protease | Bold et al. 1998 J Med Chem. 41(18):3387-401 | NIH AIDS Reagent Program, Division of AIDS, NIAID |
| **Zidovudine (AZT)** | Small molecule inhibitor | Nucleoside analogue | Mitsuya et al. 1985 Proc Natl Acad Sci U S A. 82(20): 7096–7100 | NIH AIDS Reagent Program, Division of AIDS, NIAID |
|  |  |  |  |  |
