# Supplementary material for: Capacity of Broadly Neutralizing Antibodies to Inhibit HIV-1 Cell-Cell Transmission Is Strain- and Epitope-Dependent
Source: PLoS Pathog. 2015 Jul 9;11(7):e1004966. doi: 10.1371/journal.ppat.1004966 (PMC4497647; doi:10.1371/journal.ppat.1004966)
Supplement: S3 Table — IC50 of free virus and cell-cell inhibition and fold changes IC50 between the two transmission modes for all sensitive bnAb-virus combinations. Where IC50 for cell-cell transmission could not be determined in the probed concentration range, the highest bnAb-concentration tested is indicated (bold letters). For the calculation of the fold change IC50, the IC50 of insensitive bnAb-virus combinations was nominally set to a value of two times the highest ineffective bnAb-concentration tested. These values are indicated in bold letters. (DOCX) [file ppat.1004966.s003.docx]

| **IC50 Free virus** | | | | | | | | | | | | |  |  |  |
| --- | --- | --- | --- | --- | --- | --- | --- | --- | --- | --- | --- | --- | --- | --- | --- |
|  | **Subtype B** | | | | | | | **Subtype A** | | **Subtype C** | | | |  | |
|  | **JR-FL** | **JR-CSF** | **SF162** | **DH123** | **PVO.4** | **REJO** | **THRO** | **BG505** | **BG505 N332** | **ZM53** | **ZM109** | **ZM214** | | **Breadth** | **Median** |
| **b12** | 0.007 | 0.009 | 0.008 | 0.035 |  | 4.484 | 0.041 |  |  |  |  | 0.017 | | 7 | 0.017 |
| **VRC01** | 0.017 | 0.188 | 0.318 | 0.193 | 0.357 | 0.046 | 1.533 | 0.135 | 0.050 | 1.600 | 0.168 | 0.171 | | 12 | 0.180 |
| **NIH45-46** | 0.007 | 0.033 | 0.026 | 0.060 |  | 0.003 | 0.259 | 0.014 | 0.003 |  |  | 0.040 | | 9 | 0.026 |
| **PGV04** | 0.039 | 0.056 | 0.024 | 0.049 | 0.211 | 0.025 |  | 0.023 | 0.036 | 0.841 | 0.023 | 0.344 | | 11 | 0.039 |
| **3BNC117** | 0.002 | 0.013 | 0.015 | 0.041 | 0.028 | 0.011 | 0.226 | 0.010 | 0.012 | 0.133 | 0.102 | 0.071 | | 12 | 0.021 |
| **PGT121** | 0.049 | 0.044 | 0.002 | 0.003 | 0.161 | 33.100 |  | 0.257 | 0.006 | 0.005 |  | 0.368 | | 10 | 0.046 |
| **PGT125** | 0.017 | 0.003 | 0.001 | 0.009 | 0.020 |  |  | 0.008 | 0.002 |  |  |  | | 7 | 0.008 |
| **PGT128** | 0.164 | 0.019 | 0.026 | 0.184 | 0.047 |  |  | 1.928 | 0.002 |  |  |  | | 7 | 0.047 |
| **PGT135** |  | 0.042 | 0.016 |  |  |  |  |  |  |  |  |  | | 2 | 0.029 |
| **PGT145** |  | 0.001 |  | 0.006 | 0.167 | 0.000 | 0.007 | 0.034 | 0.004 | 1.832 | 0.104 |  | | 9 | 0.007 |
| **PG9** |  | 0.007 |  | 0.181 |  | 0.015 |  | 0.045 | 0.016 | 0.092 | 0.437 |  | | 7 | 0.045 |
| **PG16** |  | 0.000 |  | 2.128 |  | 0.105 | 5.065 | 0.021 | 0.007 | 0.010 |  |  | | 7 | 0.021 |
| **2G12** | 0.411 | 0.889 | 0.436 |  | 0.880 |  |  |  | 0.467 |  |  |  | | 5 | 0.467 |
| **2F5** | 0.103 | 0.040 | 0.228 | 1.239 |  | 0.034 | 10.770 | 0.310 | 0.015 |  |  |  | | 8 | 0.165 |
| **10E8** | 0.020 | 0.016 | 0.017 | 0.073 | 0.308 | 0.010 | 0.019 | 0.035 | 0.014 | 0.343 | 0.035 | 0.093 | | 12 | 0.027 |
| **4E10** | 0.444 | 0.651 | 1.872 | 1.405 |  | 0.201 | 0.090 | 0.215 | 0.125 | 2.687 | 0.145 |  | | 10 | 0.329 |
| **T20** | 0.004 | 0.002 | 0.012 | 0.006 | 0.020 | 0.016 | 0.013 | 0.003 | 0.002 | 0.011 | 0.002 | 0.012 | | 12 | 0.008 |
| **CAP256 VRC26.08** |  |  |  |  | 52.960 |  |  | 0.142 | 0.037 | 0.004 |  | 22.980 | | 5 | 0.142 |
| **CAP256 VRC26.09** |  |  |  |  |  |  |  | 0.102 | 0.028 | 0.002 |  | 15.380 | | 4 | 0.065 |

| **IC50 Cell-cell** | | | | | | | | | | | |  |  |  |  |  |  |
| --- | --- | --- | --- | --- | --- | --- | --- | --- | --- | --- | --- | --- | --- | --- | --- | --- | --- |
|  | **Subtype B** | | | | | | | **Subtype A** | | **Subtype C** | | | |  | |  |  |
|  | **JR-FL** | **JR-CSF** | **SF162** | **DH123** | **PVO.4** | **REJO** | **THRO** | **BG505** | **BG505 N332** | **ZM53** | **ZM109** | | **ZM214** | **Breadth** | **Median** | |  |
| **b12** | 0.439 | 5.058 | **>1** | **>1** |  | 370.500 | 82.880 |  |  |  |  | | **>30** | 4 | 43.969 | |  |
| **VRC01** | 0.798 | 4.261 | 6.084 | 7.771 | 9.872 | 2.131 | **>100** | 2.521 | 1.080 | 31.280 | 2.659 | | **>40** | 10 | 3.460 | |  |
| **NIH45-46** | 0.250 | 0.568 | 0.695 | 0.875 |  | 0.100 | 29.910 | 0.633 | 0.383 |  |  | | 3.344 | 9 | 0.633 | |  |
| **PGV04** | 0.903 | 0.593 | 0.775 | 0.698 | 2.328 | 0.526 |  | 0.173 | 0.148 | 11.320 | 0.299 | | **>10** | 10 | 0.645 | |  |
| **3BNC117** | 0.083 | 0.357 | 0.841 | 1.056 | 0.938 | 0.397 | 53.370 | 0.445 | 0.283 | 3.595 | 0.673 | | **>5** | 11 | 0.673 | |  |
| **PGT121** | 1.091 | 0.959 | 0.199 | 0.083 | 2.912 | 45.860 |  | 1.726 | 0.281 | 0.106 |  | | **>10** | 9 | 0.959 | |  |
| **PGT125** | 0.344 | 0.084 | 0.126 | 0.321 | 0.420 |  |  | 0.192 | 0.112 |  |  | |  | 7 | 0.192 | |  |
| **PGT128** | 2.638 | 0.438 | 0.575 | 2.318 | 1.069 |  |  | 2.799 | 0.070 |  |  | |  | 7 | 1.069 | |  |
| **PGT135** |  | 0.435 | 0.313 |  |  |  |  |  |  |  |  | |  | 2 | 0.374 | |  |
| **PGT145** |  | 0.016 |  | 0.165 | 3.260 | 0.036 | 0.138 | 0.088 | 0.037 | 0.149 | 0.256 | |  | 9 | 0.138 | |  |
| **PG9** |  | 0.101 |  | 1.639 |  | 0.812 |  | 0.981 | 0.351 | 1.055 | 7.040 | |  | 7 | 0.981 | |  |
| **PG16** |  | 0.023 |  | 0.560 |  | 6.272 | 2.802 | 0.199 | 0.151 | 0.112 |  | |  | 7 | 0.199 | |  |
| **2G12** | 1.834 | 1.842 | 8.611 |  | 6.312 |  |  |  | 0.357 |  |  | |  | 5 | 1.842 | |  |
| **2F5** | 1.621 | 0.631 | 3.326 | 12.200 |  | 1.122 | **>100** | 1.509 | 0.508 |  |  | |  | 7 | 1.509 | |  |
| **10E8** | 0.244 | 0.407 | 0.464 | 2.181 | 2.893 | 0.199 | 0.573 | 0.344 | 0.288 | 0.232 | 0.342 | | 0.817 | 12 | 0.376 | |  |
| **4E10** | 8.459 | 2.985 | 16.620 | 62.630 |  | 2.669 | 8.969 | 5.808 | 2.614 | 2.386 | 6.381 | |  | 10 | 6.095 | |  |
| **T20** | 0.013 | 0.004 | 0.080 | 0.021 | 0.129 | 0.019 | 0.022 | 0.002 | 0.002 | 0.029 | 0.016 | | 0.062 | 12 | 0.020 | |  |
| **CAP256 VRC26.08** |  |  |  |  | 39.990 |  |  | 0.556 | 0.264 | 0.033 |  | | 2.086 | 5 | 0.556 | |  |
| **CAP256 VRC26.09** |  |  |  |  |  |  |  | 0.144 | 0.098 | 0.020 |  | | 0.645 | 4 | 0.121 | | |

| **Change inhibitory potency cell-cell (Fold change IC50)** | | | | | | | | | | | |  |  |  |
| --- | --- | --- | --- | --- | --- | --- | --- | --- | --- | --- | --- | --- | --- | --- |
|  | **Subtype B** | | | | | | | **Subtype A** | | **Subtype C** | | | |  |
|  | **JR-FL** | **JR-CSF** | **SF162** | **DH123** | **PVO.4** | **REJO** | **THRO** | **BG505** | **BG505 N332** | **ZM53** | **ZM109** | | **ZM214** | **Median** |
| **b12** | 61 | 595 | **256** | **57** |  | 83 | 2038 |  |  |  |  | | **3540** | 256 |
| **VRC01** | 48 | 23 | 19 | 40 | 28 | 47 | **130** | 19 | 21 | 20 | 16 | | **234** | 25 |
| **NIH45-46** | 35 | 17 | 27 | 15 |  | 36 | 116 | 45 | 121 |  |  | | 84 | 36 |
| **PGV04** | 23 | 11 | 33 | 14 | 11 | 21 |  | 7.5 | 4.1 | 13 | 13 | | **29** | 13 |
| **3BNC117** | 39 | 27 | 57 | 26 | 34 | 35 | 237 | 43 | 23 | 27 | 6.6 | | **142** | 34 |
| **PGT121** | 22 | 22 | 82 | 28 | 18 | 1.4 |  | 6.7 | 51 | 19 |  | | **54** | 22 |
| **PGT125** | 20 | 29 | 95 | 35 | 21 |  |  | 25 | 47 |  |  | |  | 29 |
| **PGT128** | 16 | 23 | 22 | 13 | 23 |  |  | 1.5 | 46 |  |  | |  | 22 |
| **PGT135** |  | 10 | 19 |  |  |  |  |  |  |  |  | |  | 15 |
| **PGT145** |  | 19 |  | 29 | 19 | 79 | 20 | 2.6 | 10 | 0.1 | 2.5 | |  | 19 |
| **PG9** |  | 14 |  | 9.0 |  | 53 |  | 22 | 22 | 11 | 16 | |  | 16 |
| **PG16** |  | 80 |  | 0.3 |  | 60 | 0.6 | 10 | 21 | 11 |  | |  | 11 |
| **2G12** | 4.5 | 2.1 | 20 |  | 7.2 |  |  |  | 0.8 |  |  | |  | 4.5 |
| **2F5** | 16 | 16 | 15 | 10 |  | 33 | **19** | 4.9 | 33 |  |  | |  | 16 |
| **10E8** | 12 | 25 | 28 | 30 | 9.4 | 20 | 31 | 10 | 21 | 0.7 | 10 | | 8.8 | 16 |
| **4E10** | 19 | 4.6 | 8.9 | 45 |  | 13 | 100 | 27 | 21 | 0.9 | 44 | |  | 20 |
| **T20** | 3.0 | 2.3 | 6.6 | 3.7 | 6.5 | 1.2 | 1.6 | 0.8 | 0.9 | 2.6 | 8.8 | | 5.3 | 2.8 |
| **CAP256 VRC26.08** |  |  |  |  | 0.8 |  |  | 3.9 | 7.0 | 8.7 |  | | 0.1 | 3.9 |
| **CAP256 VRC26.09** |  |  |  |  |  |  |  | 1.4 | 3.5 | 10 |  | | 0.0 | 2.5 |
