# Supplementary material for: Capacity of Broadly Neutralizing Antibodies to Inhibit HIV-1 Cell-Cell Transmission Is Strain- and Epitope-Dependent
Source: PLoS Pathog. 2015 Jul 9;11(7):e1004966. doi: 10.1371/journal.ppat.1004966 (PMC4497647; doi:10.1371/journal.ppat.1004966)
Supplement: S4 Table — Slope values, m, were determined from free virus and cell-cell inhibition assays for all bnAb-virus combinations (S5A–S5P Fig) by fitting the Hill curve equation cm/(cm +IC50 m) to the inhibition data. Parameters are shown for all bnAb-virus combinations for which this fitting procedure was successful. (DOCX) [file ppat.1004966.s004.docx]

| **Slope values Free virus** | | | | | | | | | | | |  |  |
| --- | --- | --- | --- | --- | --- | --- | --- | --- | --- | --- | --- | --- | --- |
|  | **Subtype B** | | | | | | | **Subtype A** | | **Subtype C** | | | |
|  | **JR-FL** | **JR-CSF** | **SF162** | **DH123** | **PVO.4** | **REJO** | **THRO** | **BG505** | **BG505 N332** | **ZM53** | **ZM109** | | **ZM214** |
| **b12** | 0.816 | 0.418 |  |  |  | 0.454 | 0.478 |  |  |  |  | |  |
| **VRC01** | 0.656 | 0.825 | 1.111 | 0.725 | 0.858 | 0.789 |  | 1.499 | 1.053 | 1.143 | 0.731 | |  |
| **NIH45-46** | 0.804 | 0.814 | 0.777 | 0.868 |  | 0.767 | 0.702 | 0.604 | 0.471 |  |  | | 0.569 |
| **PGV04** | 0.836 | 1.066 | 0.862 | 0.715 | 0.969 | 1.032 |  | 1.201 | 1.772 | 1.053 | 1.061 | | 1.034 |
| **3BNC117** | 0.921 | 0.703 | 0.694 | 1.054 | 1.060 | 0.652 | 0.527 | 0.668 | 1.326 | 0.792 | 0.748 | |  |
| **PGT121** | 0.888 | 0.890 | 0.674 | 0.836 | 0.768 | 0.749 |  | 0.602 | 0.783 | 0.830 |  | | 0.802 |
| **PGT125** | 1.099 | 0.868 | 0.673 | 0.896 | 0.612 |  |  | 1.155 | 0.835 |  |  | |  |
| **PGT128** | 0.637 | 0.786 | 0.739 | 0.983 | 0.535 |  |  | 0.784 | 0.681 |  |  | |  |
| **PGT135** |  | 0.829 | 0.601 |  |  |  |  |  |  |  |  | |  |
| **PGT145** |  | 0.702 |  | 0.839 | 0.758 | 0.632 | 0.874 | 0.670 | 0.380 | 0.629 | 0.378 | |  |
| **PG9** |  | 0.980 |  | 0.449 |  | 0.407 | 0.503 | 0.893 | 0.761 | 0.930 | 0.430 | |  |
| **PG16** |  | 0.404 |  | 0.259 |  | 0.183 | 0.351 | 0.780 | 0.646 | 0.387 |  | |  |
| **2G12** | 0.741 | 0.871 | 0.480 |  | 0.984 |  |  |  | 0.651 |  |  | |  |
| **2F5** | 0.693 | 0.549 | 0.699 | 0.659 |  | 0.500 | 0.718 | 0.632 | 0.431 |  |  | |  |
| **10E8** | 0.771 | 0.501 | 0.504 | 0.523 | 0.816 | 0.514 | 0.678 | 0.751 | 0.638 | 1.016 | 0.657 | | 0.498 |
| **4E10** | 0.719 | 0.766 | 0.774 | 0.549 |  | 0.617 | 0.486 | 0.468 | 0.685 | 1.139 | 0.597 | |  |
| **T20** | 1.789 | 1.187 | 1.455 | 1.744 | 1.257 | 2.225 | 1.150 | 1.820 | 2.525 | 1.029 | 0.862 | | 0.744 |

| **Slope values Cell-cell** | | | | | | | | | | | |  |  |
| --- | --- | --- | --- | --- | --- | --- | --- | --- | --- | --- | --- | --- | --- |
|  | **Subtype B** | | | | | | | **Subtype A** | | **Subtype C** | | | |
|  | **JR-FL** | **JR-CSF** | **SF162** | **DH123** | **PVO.4** | **REJO** | **THRO** | **BG505** | **BG505 N332** | **ZM53** | **ZM109** | | **ZM214** |
| **b12** | 1.117 | 0.703 |  |  |  | 0.471 | 1.642 |  |  |  |  | |  |
| **VRC01** | 0.778 | 1.170 | 1.088 | 1.025 | 1.120 | 1.081 |  | 1.221 | 1.094 | 1.063 | 0.626 | |  |
| **NIH45-46** | 1.025 | 0.746 | 1.110 | 1.562 |  | 0.680 | 0.800 | 1.292 | 1.158 |  |  | | 0.820 |
| **PGV04** | 0.888 | 0.883 | 1.306 | 0.875 | 1.088 | 1.196 |  | 0.542 | 0.687 | 0.919 | 0.520 | | 0.335 |
| **3BNC117** | 1.024 | 1.174 | 1.153 | 1.078 | 1.423 | 0.618 | 1.794 | 1.055 | 1.053 | 1.100 | 0.450 | |  |
| **PGT121** | 1.300 | 1.042 | 1.469 | 1.173 | 1.533 | 1.007 |  | 1.000 | 1.016 | 1.064 |  | | 0.106 |
| **PGT125** | 0.864 | 0.945 | 1.155 | 1.160 | 1.223 |  |  | 1.211 | 1.541 |  |  | |  |
| **PGT128** | 1.158 | 1.107 | 1.265 | 1.219 | 1.315 |  |  | 1.518 | 0.814 |  |  | |  |
| **PGT135** |  | 0.792 | 0.792 |  |  |  |  |  |  |  |  | |  |
| **PGT145** |  | 0.749 |  | 1.151 | 1.741 | 1.466 | 1.325 | 0.832 | 0.811 | 0.482 | 0.386 | |  |
| **PG9** |  | 0.975 |  | 0.976 |  | 0.556 | 0.907 | 0.989 | 0.686 | 1.201 | 0.425 | |  |
| **PG16** |  | 0.600 |  | 0.538 |  | 0.315 | 0.460 | 0.609 | 0.964 | 0.676 |  | |  |
| **2G12** | 0.705 | 0.618 | 0.774 |  | 0.920 |  |  |  | 0.613 |  |  | |  |
| **2F5** | 1.105 | 0.749 | 1.091 | 1.132 |  | 1.128 | 0.333 | 0.861 | 0.724 |  |  | |  |
| **10E8** | 0.982 | 1.422 | 0.851 | 1.255 | 1.619 | 0.905 | 1.089 | 0.964 | 0.847 | 0.585 | 0.558 | | 0.808 |
| **4E10** | 0.941 | 0.439 | 0.956 | 0.980 |  | 0.681 | 0.893 | 0.841 | 0.552 | 0.808 | 0.534 | |  |
| **T20** | 1.307 | 0.932 | 1.417 | 1.616 | 1.558 | 1.282 | 1.218 | 1.368 | 1.194 | 0.736 | 0.896 | | 1.069 |
