# Supplementary material for: Capacity of Broadly Neutralizing Antibodies to Inhibit HIV-1 Cell-Cell Transmission Is Strain- and Epitope-Dependent
Source: PLoS Pathog. 2015 Jul 9;11(7):e1004966. doi: 10.1371/journal.ppat.1004966 (PMC4497647; doi:10.1371/journal.ppat.1004966)
Supplement: S5 Table — Maximal concentrations used for the assays determining pre- and post-attachment and total activity (Figs 6, 7 and 8) and maximal percentage of neutralization obtained are indicated. (DOCX) [file ppat.1004966.s005.docx]

| **Maximal concentrations used [µg/ml]** | | | | | | | | |
| --- | --- | --- | --- | --- | --- | --- | --- | --- |
|  | **Subtype B** | | **Subtype A** | | **Subtype C** | | |  |
|  | **JR-FL** | **JR-CSF** | **BG505** | **BG505 N332** | **ZM53** | **ZM109** | **ZM214** |  |
| **b12** | 50 | 50 |  |  |  |  | 50 |  |
| **VRC01** | 50 | 100 | 20 | 20 | 100 | 50 | 50 |  |
| **NIH45-46** | 10 | 20 | 20 | 20 |  |  | 30 |  |
| **PGV04** | 20 | 20 | 20 | 20 | 50 | 10 | 50 |  |
| **3BNC117** | 5 | 10 | 20 | 20 | 50 | 50 | 10 |  |
| **PGT121** | 10 | 10 | 50 | 50 | 15 |  | 30 |  |
| **PGT125** | 20 | 10 | 30 | 30 |  |  |  |  |
| **PGT128** | 5 | 10 | 50 | 50 |  |  |  |  |
| **PGT135** |  | 50 |  |  |  |  |  |  |
| **PGT145** |  | 10 | 50 | 50 | 100 | 50 |  |  |
| **PG9** |  | 10 | 20 | 20 | 100 | 50 |  |  |
| **PG16** |  | 100 | 50 | 50 | 100 |  |  |  |
| **2G12** | 100 | 100 |  | 100 |  |  |  |  |
| **2F5** | 100 | 100 | 100 | 100 |  |  |  |  |
| **10E8** | 10 | 20 | 20 | 20 | 30 | 20 | 30 |  |
| **4E10** | 100 | 100 | 100 | 100 | 100 | 100 |  |  |
| **T20** | 1 | 2 | 1 | 1 | 5 | 1 | 1 |  |
| **CAP256 VRC26.08** |  |  | 50 | 50 | 2 |  | 100 |  |
| **CAP256 VRC26.09** |  |  | 50 | 50 | 2 |  | 100 |  |

| **Maximal neutralization [%]** | | | | | | | | |
| --- | --- | --- | --- | --- | --- | --- | --- | --- |
|  | **Subtype B** | | **Subtype A** | | **Subtype C** | | |  |
|  | **JR-FL** | **JR-CSF** | **BG505** | **BG505 N332** | **ZM53** | **ZM109** | **ZM214** |  |
| **b12** | 100.0 | 100.0 |  |  |  |  | 72.1 |  |
| **VRC01** | 100.0 | 100.0 | 100.0 | 99.9 | 97.8 | 100.0 | 98.8 |  |
| **NIH45-46** | 99.9 | 100.0 | 99.9 | 100.0 |  |  | 99.9 |  |
| **PGV04** | 99.4 | 100.0 | 99.8 | 100.0 | 92.9 | 100.0 | 99.8 |  |
| **3BNC117** | 100.0 | 100.0 | 100.0 | 100.0 | 100 | 95.0 | 98.6 |  |
| **PGT121** | 100.0 | 100.0 | 88.6 | 99.9 | 98.9 |  | 96.6 |  |
| **PGT125** | 99.8 | 100.0 | 99.7 | 100.0 |  |  |  |  |
| **PGT128** | 100.0 | 100.0 | 98.8 | 99.9 |  |  |  |  |
| **PGT135** |  | 98.3 |  |  |  |  |  |  |
| **PGT145** |  | 100.0 | 96.3 | 98.8 | 99.2 | 82.4 |  |  |
| **PG9** |  | 99.5 | 100.0 | 100.0 | 93.5 | 67.1 |  |  |
| **PG16** |  | 98.4 | 97.7 | 99.9 | 85.9 |  |  |  |
| **2G12** | 93.1 | 96.2 |  | 93.8 |  |  |  |  |
| **2F5** | 100.0 | 100.0 | 100.0 | 100.0 |  |  |  |  |
| **10E8** | 99.9 | 100.0 | 99.9 | 99.9 | 99.9 | 100.0 | 97.9 |  |
| **4E10** | 98.3 | 99.6 | 100.0 | 100.0 | 100.0 | 97.2 |  |  |
| **T20** | 100.0 | 100.0 | 100.0 | 100.0 | 100.0 | 99.9 | 98.0 |  |
| **CAP256 VRC26.08** |  |  | 84.4 | 92.6 | 99.8 |  | 51.8 |  |
| **CAP256 VRC26.09** |  |  | 75.0 | 94.2 | 100.0 |  | 50.3 |  |
