# Supplementary figures and images for: Capacity of Broadly Neutralizing Antibodies to Inhibit HIV-1 Cell-Cell Transmission Is Strain- and Epitope-Dependent
Source: PLoS Pathog. 2015 Jul 9;11(7):e1004966. doi: 10.1371/journal.ppat.1004966 (PMC4497647; doi:10.1371/journal.ppat.1004966)

## A b12

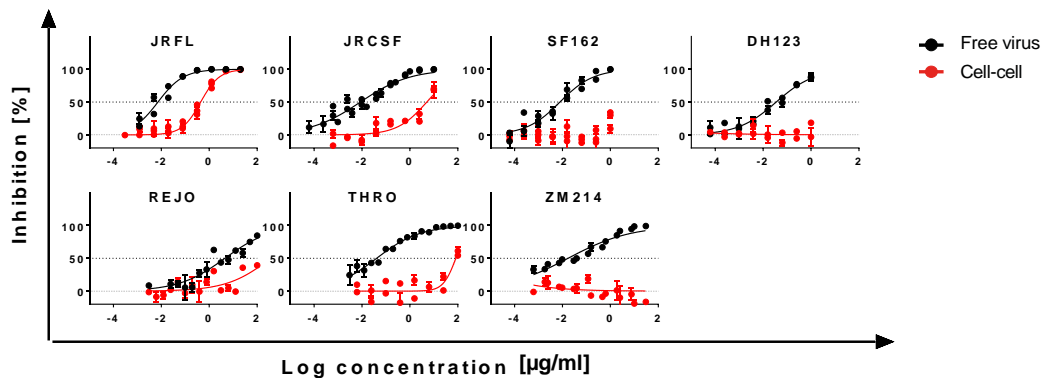

## B VRC01

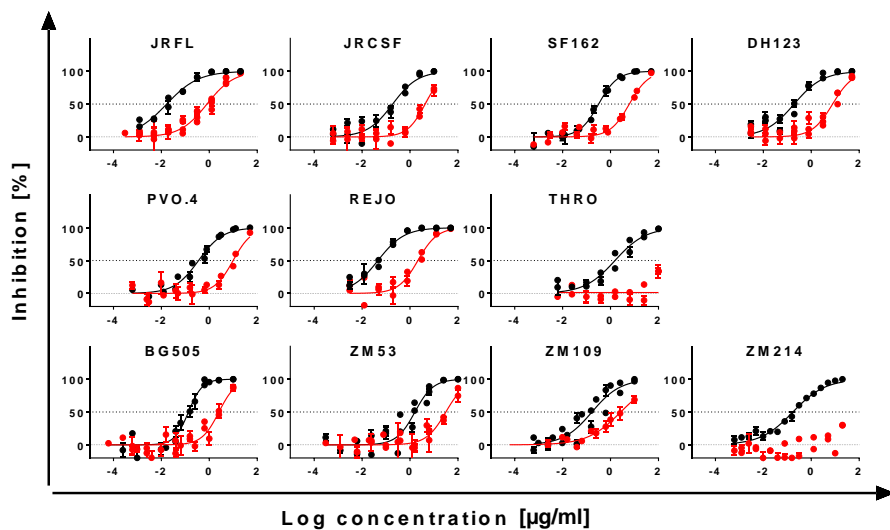

## C PGV04

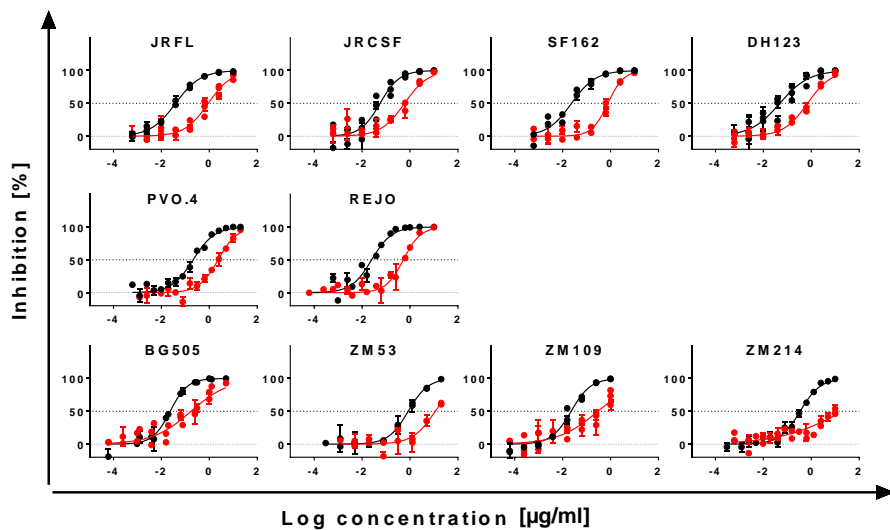

## D NIH45-46

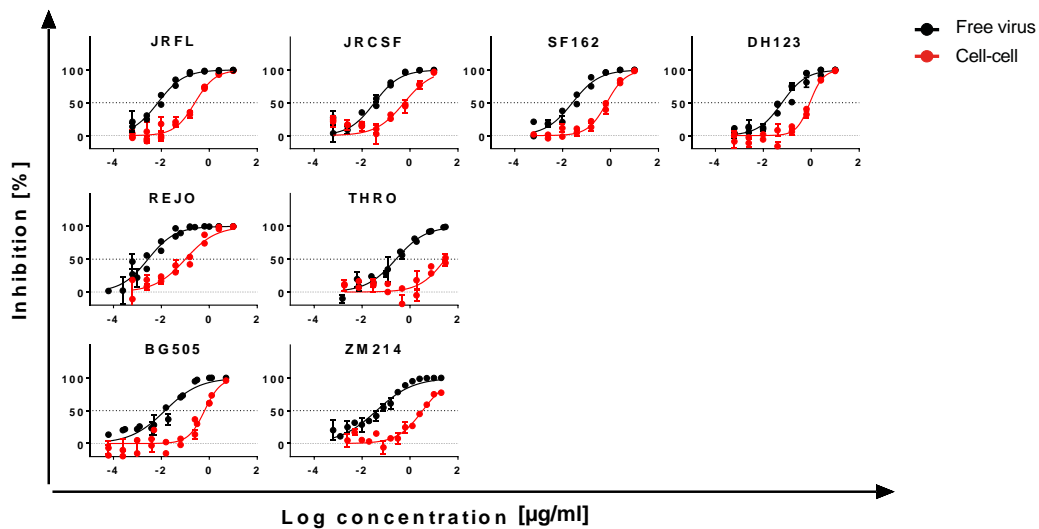

## E 3BNC117

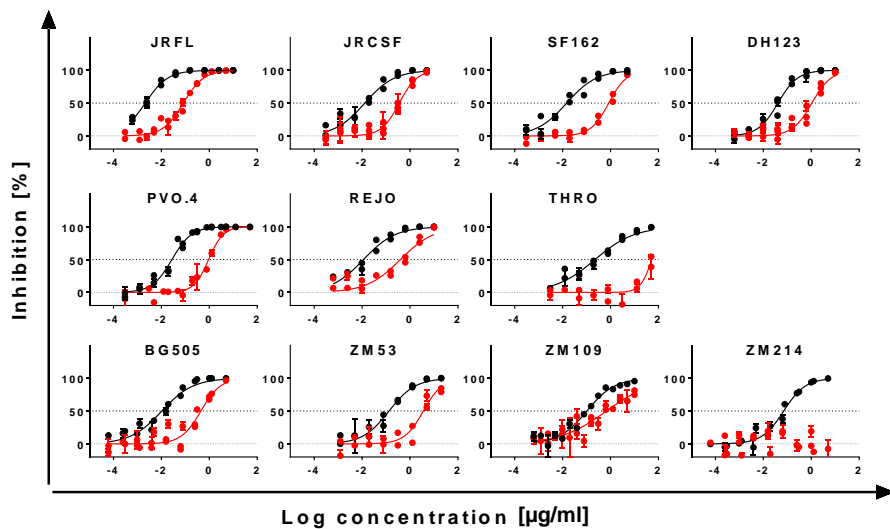

## F PGT121

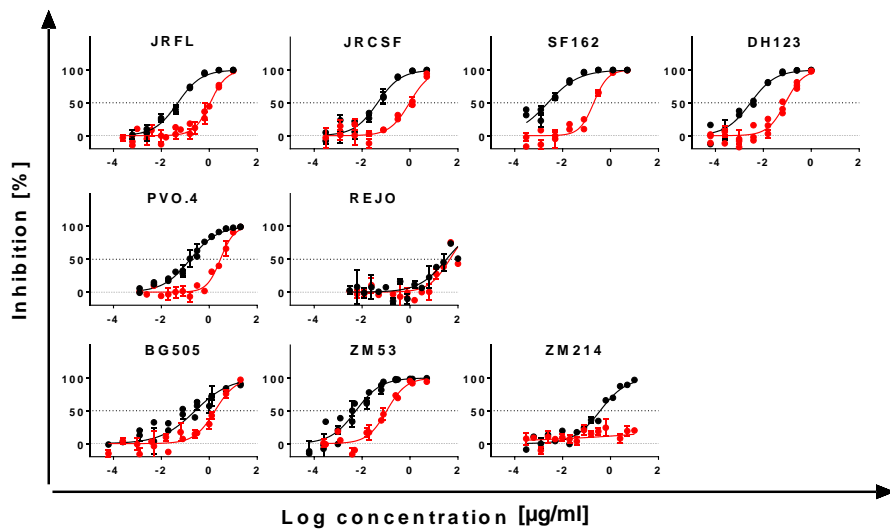

**G PGT125**

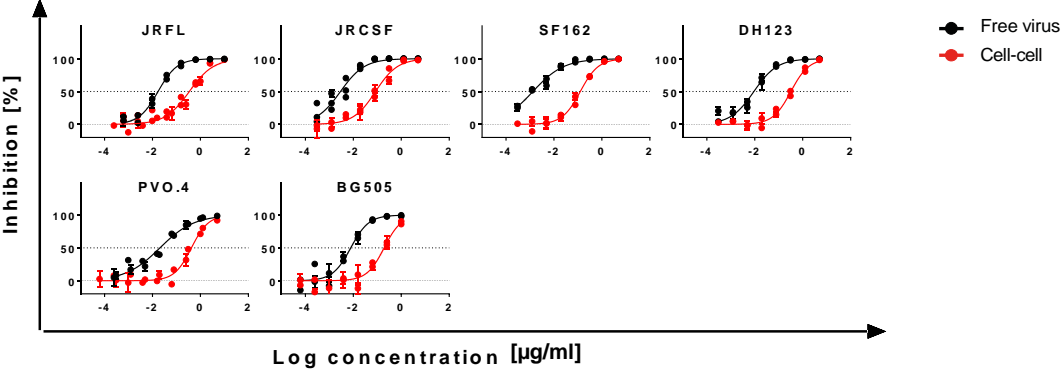

**H PGT128**

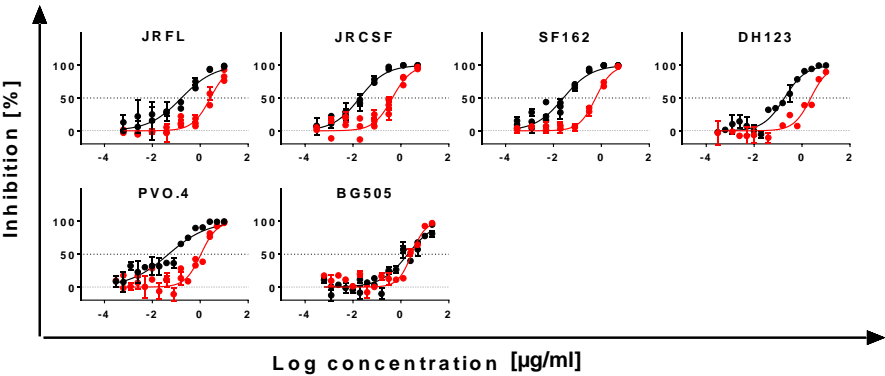

**I PGT135**

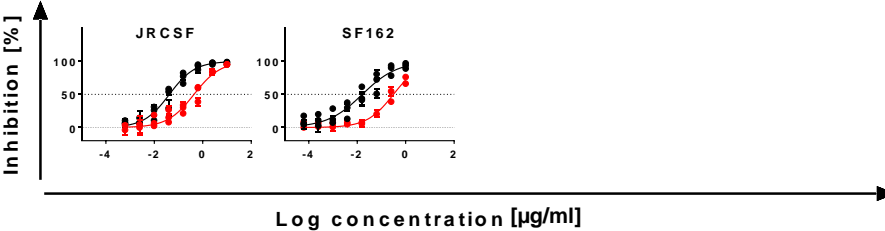

## J PGT145

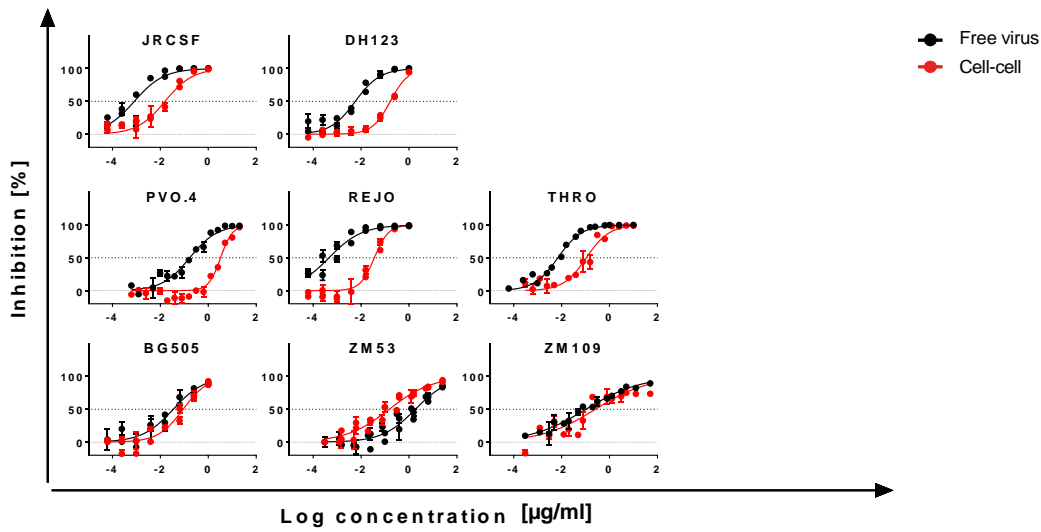

## K PG9

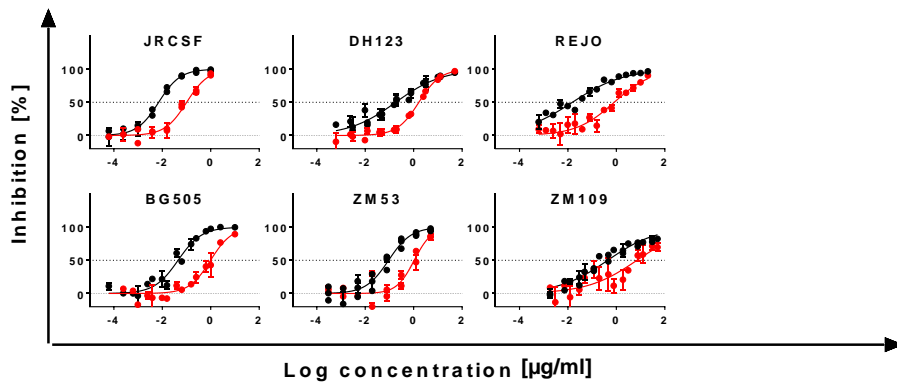

## L PG16

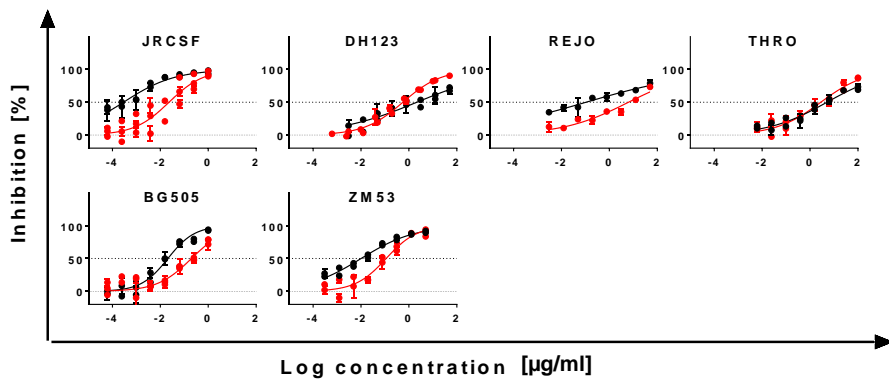

## M 2G12

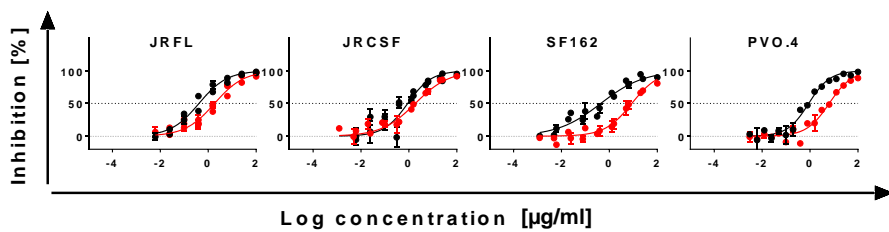

**N 2F5**

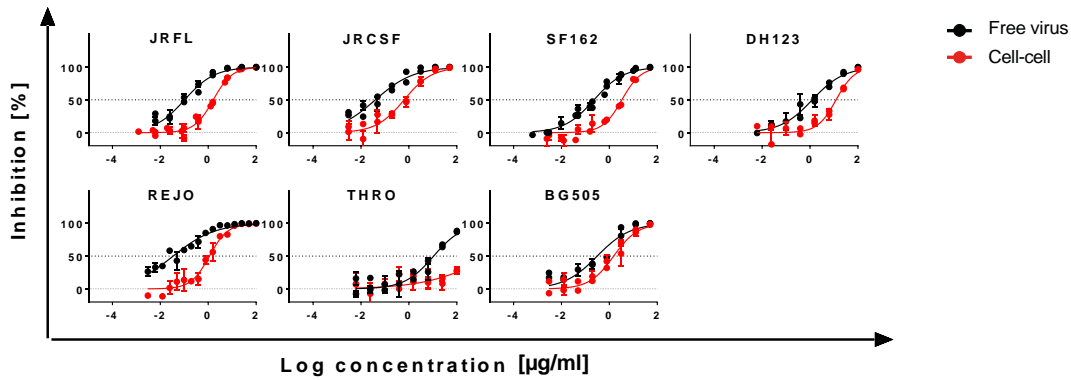

**O 10E8**

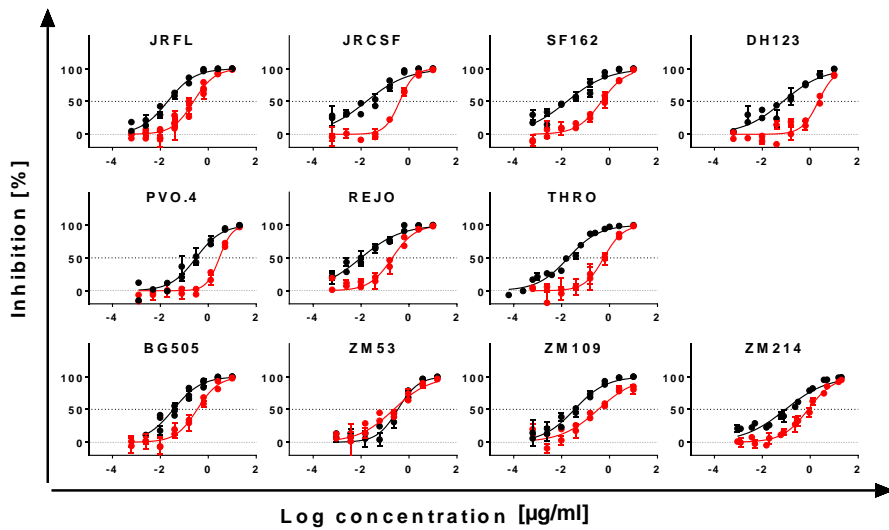

**P 4E10**

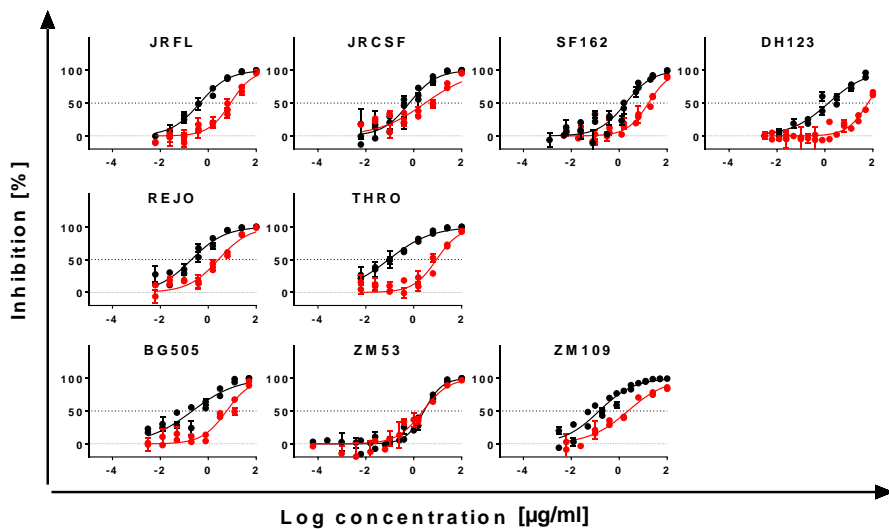

Supplement: S5 Fig — A-P: Addendum to Fig 2. Inhibition of free virus (black circles) and cell-cell (red circles) transmission of subtype A, B and C virus strains by the indicated bnAbs was studied. The graphs show means and standard error of means (SEM, error bars) of two to three independent experiments performed in duplicates and curve fits to sigmoid dose response curves (variable slope). Subfigures A-P show free virus and cell-cell inhibition profiles determined for each bnAb. (PDF) [file ppat.1004966.s010.pdf]
